# Genotype-Associated Molecular Phenotypes Across Brain and Gut Compartments in Male 5xFAD Mice: Shared Latent Structure and the Limits of Individual-Level Inference

**DOI:** 10.3390/ijms27219879

**Published:** 2026-11-04

**Authors:** Betul Sahin, Yunus Eken, Furkan Sahin, Tayyip Karaman, Osman Uğur Sezerman, Samed Ozer, Zelal Zuhal Kaya, Mustafa Zafer Karagözlü, Muhittin Abdulkadir Serdar, Mustafa Serteser, Ahmet Tarık Baykal

**Affiliations:** 1 Department of Medical Biology, School of Medicine, Bahçeşehir University, Istanbul 34734, Türkiye; betul.sahin1@bau.edu.tr; 2 Department of Translational Medicine, Graduate School of Health Sciences, Acibadem Mehmet Ali Aydinlar University, Istanbul 34752, Türkiye; yunusekn5@gmail.com; 3 Department of Biochemistry and Molecular Biology, Graduate School of Health Sciences, Acibadem Mehmet Ali Aydinlar University, Istanbul 34752, Türkiye; furkan.sahin1@live.acibadem.edu.tr; 4 Acibadem Labmed Clinical Laboratories, Istanbul 34752, Türkiye; 5 Department of Basic Medical Sciences, Faculty of Medicine, Nakhchivan State University, Nakhchivan AZ7012, Azerbaijan; tayyipkaramannn@gmail.com; 6 Department of Biostatistics and Medical Informatics, School of Medicine, Acibadem Mehmet Ali Aydinlar University, Istanbul 34752, Türkiye; ugur.sezerman@acibadem.edu.tr; 7 Experimental Animals Application and Research Center, Acibadem Mehmet Ali Aydinlar University, Istanbul 34752, Türkiye; samed.ozer@acibadem.edu.tr; 8 Department of Medical Biochemistry, Faculty of Medicine, Nişantaşı University, Istanbul 34398, Türkiye; zelalzuhal.kaya@nisantasi.edu.tr; 9 Department of Molecular Biology and Genetics, Faculty of Arts and Sciences, Haliç University, Istanbul 34060, Türkiye; zaferkaragozlu@halic.edu.tr; 10 Department of Medical Biochemistry, School of Medicine, Acibadem Mehmet Ali Aydinlar University, Istanbul 34752, Türkiye; muhittin.serdar@acibademlabmed.com.tr (M.A.S.); mserteser@outlook.com (M.S.)

**Keywords:** Alzheimer’s disease, 5xFAD, microbiota–gut–brain axis, multi-omics factor analysis, proteomics, shotgun metagenomics, *Turicibacter*, permutation inference

## Abstract

Whether gut and brain molecular states covary within individual animals, rather than merely co-occurring at the group level, has rarely been tested in Alzheimer’s disease models. We profiled the hippocampus, cortex, cerebellum, intestine and feces of 18 male 5xFAD (5 familial AD mutations) (B6SJL background) mice and wild-type littermates at 3, 6 and 9 months by label-free proteomics, and feces additionally by shotgun metagenomics. The hippocampal proteome reproduced the amyloid-associated signature (App, Apoe, Hexb, Clu, Gfap), and protein-set analysis showed elevated microglial, lysosomal, astrocytic and endoplasmic reticulum chaperone proteins in the hippocampus and cortex, with no detectable difference in synaptic or mitochondrial sets. Fecal Shannon diversity was reduced, and the bile-acid-modifying genera *Romboutsia* and *Turicibacter* were nearly absent in all 5xFAD animals from 3 months (cage nested within genotype). Multi-omics factor analysis recovered a genotype-associated factor shared across brain and gut views (exact permutation *p* = 0.00125) that explained at most 6.3% of variance in any view and was preprocessing-dependent. After removal of group structure, correlation burden did not exceed a within-cell permutation null, and neither did same-animal positive controls. The 5xFAD genotype thus alters every compartment examined, and a modest latent structure links them; because the design excludes only strong or dense individual-level coupling, the null result bounds design resolution rather than showing that gut and brain are uncoupled.

## 1. Introduction

Alzheimer’s disease (AD) is defined by cerebral amyloid-β (Aβ) deposition and neurofibrillary pathology, but the molecular changes that accompany it are not confined to the brain parenchyma. Over the past decade the microbiota–gut–brain axis has become a major organizing framework in neurodegeneration research, supported by anatomical and signaling routes that include the vagus nerve, the enteric nervous system, systemic immunity, and microbially produced or modified metabolites [1,2]. In AD specifically, the evidence has accumulated along two largely independent lines. The first is interventional. Germ-free *APPPS1* mice develop markedly less cerebral amyloid than conventionally colonized littermates, and colonization with microbiota from transgenic donors restores amyloid burden more effectively than microbiota from wild-type donors [3]. Transfer of a healthy microbiota into an amyloid-and-tau model reduced plaque and tangle burden, gliosis and cognitive impairment [4]. Probiotic treatment modified proteolytic and cognitive outcomes in 3xTg-AD mice [5], antibiotic treatment attenuated amyloidogenic processing in 5xFAD mice, and the small molecule R13 suppressed the associated gut CCAAT/enhancer-binding protein β/asparagine endopeptidase C/EBPβ/AEP signaling [6] and remodeling of the microbiota by sodium oligomannate suppressed peripheral amino acid accumulation and the associated neuroinflammation [7]. These experiments establish that the microbiota can modify amyloid pathology when it is deliberately perturbed.

The second line is observational and much larger. Fecal microbiota composition differs between people with AD and controls [8], and comparable differences have been reported across transgenic models. In 5xFAD mice, microbiome composition diverges from wild-type animals in parallel with reduced fecal tryptic activity and expression of the human amyloid precursor protein transgene in gut tissue [9]; gut barrier proteins are lost and intestinal NLRP3 inflammasome components are elevated alongside brain gliosis [10]; and a longitudinal survey found depletion of *Turicibacter* species at 18 months, although cage of origin explained a larger share of microbiome variance than genotype [11]. Fecal metabolomic changes in the same model include shifts in bile-acid-related metabolites [12], and intestinal metaproteomic profiles separate transgenic from wild-type animals [13]. In Tg2576 mice, intestinal epithelial barrier dysfunction and vascular amyloid deposition in the gut precede detectable cerebral plaques [14].

The inference that is routinely drawn from this second body of work, that the observed gut changes and the observed brain changes are two ends of a single axis, is stronger than the designs that generate it. In most observational studies the two compartments are compared between genotype groups separately, and their co-occurrence in the same animals is treated as evidence of a functional relationship. Group-level co-occurrence, however, is the expected consequence of any genotype that independently perturbs both compartments, and it carries no information about whether the compartments are coupled within an individual. The question that distinguishes the two situations is whether an animal whose gut deviates from its group mean also has a brain that deviates correspondingly. That question requires the same animals to be measured in both compartments, an explicit removal of group structure, and a null distribution against which the residual covariation can be judged. To our knowledge it has not been posed directly in an AD model, although multi-omics factor analysis (MOFA) has been used to describe shared structure across microbiome and host layers in other contexts [15].

Multi-omics factor analysis provides a principled way to ask whether heterogeneous data views share low-dimensional structure, by inferring latent factors that explain variance across views without supervision [16,17]. It is well suited to describing a multi-compartment phenotype, but a latent factor aligned with genotype is not in itself evidence of communication, for the same reason that group-level co-occurrence is not: views that each differ by genotype will tend to share a genotype-aligned axis whether or not they influence one another. Small cohorts compound the interpretive difficulty, because high-dimensional multivariate methods can generate apparently decisive separations from very few biological units unless feature selection is nested inside validation and significance is calibrated against permuted labels [18,19].

We therefore designed the present analysis to separate three claims that are often conflated. The first is that 5xFAD genotype is associated with molecular differences in brain and gut compartments, which we test compartment by compartment. The second is that these differences share a latent structure detectable by unsupervised integration, which we test with MOFA+ and calibrate by exact enumeration of all within-age genotype label assignments. The third is that gut and brain states are coupled within individual animals, which we test on group-centered residuals against a within-cell permutation null, with same-animal positive controls included to establish what the design can resolve. All measurements derive from a single cohort of 18 male mice profiled under one standardized analytical workflow, so that compartment comparisons are not confounded by separate experiments.

## 2. Results

### 2.1. Cohort, Workflow and Data Structure

**Figure 1:**
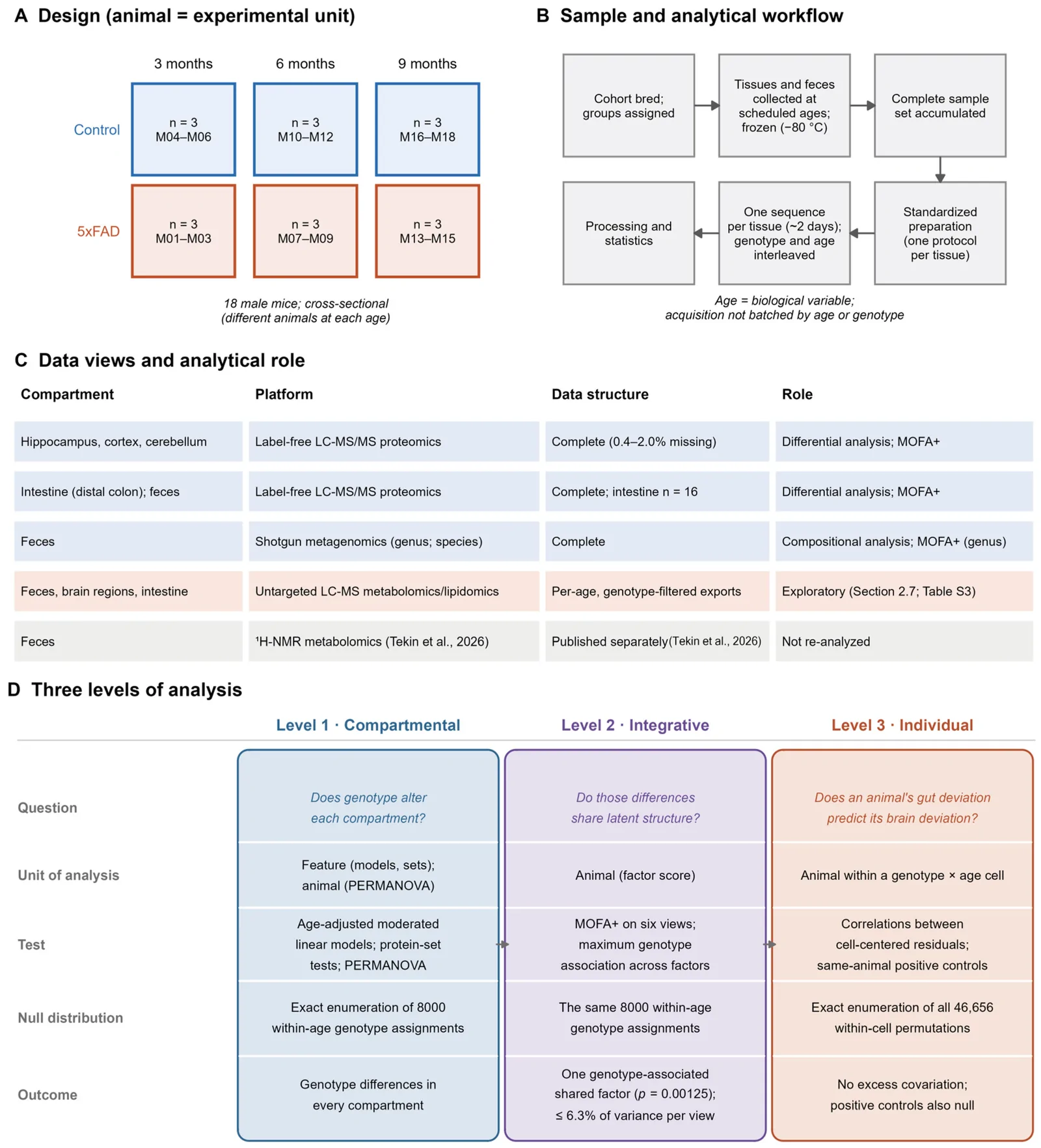
Study design and analytical workflow. (**A**) Two genotypes at three ages with three male animals per cell and different animals at each age; the animal is the experimental unit. (**B**) Sample and analytical workflow: collection at the scheduled ages, freezing, accumulation of the complete set, preparation of all samples of each tissue together, acquisition as one sequence per tissue (2 days) with genotype and age interleaved within each sequence, and subsequent computational analysis. (**C**) Data views and the analytical role assigned to each. (**D**) The three levels of analysis. Level 1, compartmental: does genotype alter each compartment? Age-adjusted moderated linear models, protein-set tests and permutational analysis of variance, calibrated by exact enumeration of 8000 within-age genotype assignments. Level 2, integrative: do the compartment-level differences share latent structure? multi-omics factor analysis (MOFA+) on six views, with the maximum genotype association across factors calibrated against the same 8000 assignments. Level 3, individual: does an animal’s gut deviation predict its brain deviation? Correlations between cell-centered residuals, calibrated by all 46,656 within-cell permutations and benchmarked against same-animal positive controls [12].

Eighteen male mice (nine 5xFAD and nine controls) were studied at 3, 6 and 9 months of age, with three animals per genotype at each age and different animals at each time point (Table 1). Tissues and fecal material were collected at the scheduled ages and stored at −80 °C until analysis. All samples were processed only after the complete sample set had been collected. All samples of each tissue were prepared together using a standardized filter-aided sample preparation (FASP) protocol and acquired as a separate sequence of approximately two days, in which the 18 samples were run in an interleaved order, with the three ages and both genotypes intermixed rather than grouped; samples from different tissues were not intermixed within a sequence (Figure 1A,B). Acquisition of the next tissue began only after the preceding sequence had been completed and instrument performance had been verified. At each age, 5xFAD mice were compared with age-matched controls.

Six data views were available at all ages and were used as the basis for integration (an initial model that also included the untargeted liquid chromatography–mass spectrometry (LC-MS) matrices is shown in Figure S6): the hippocampal, cortical, cerebellar, intestinal and fecal proteomes, and the fecal metagenome at genus level (Figure 1C). Missing values in these views ranged from 0.4% to 3.1%, except for the intestinal proteome, where two animals were absent entirely and *n* is therefore 16. Species-level metagenomic profiles were retained as a sensitivity analysis because they are largely redundant with genus-level profiles.

Untargeted LC-MS metabolomic and lipidomic matrices were also available but were excluded from the primary integrative model, because the features they contain were already filtered on the genotype contrast and are largely confined to single age blocks. This is a decision about data structure and model validity rather than a claim that the six-view model is intrinsically preferable, and the evidence for it is set out in Section 2.7. These layers are reported there descriptively. Two 6-month control hippocampal samples showed median log2 intensities 1.1 (M10) and 0.7 (M12) log2 units below the median of the remaining 16 samples (Figure S2), and all proteomes were accordingly median-normalized per sample before analysis.

### 2.2. The Brain Proteome Phenotype Is Glial and Proteostatic Rather than Bioenergetic

**Figure 2:**
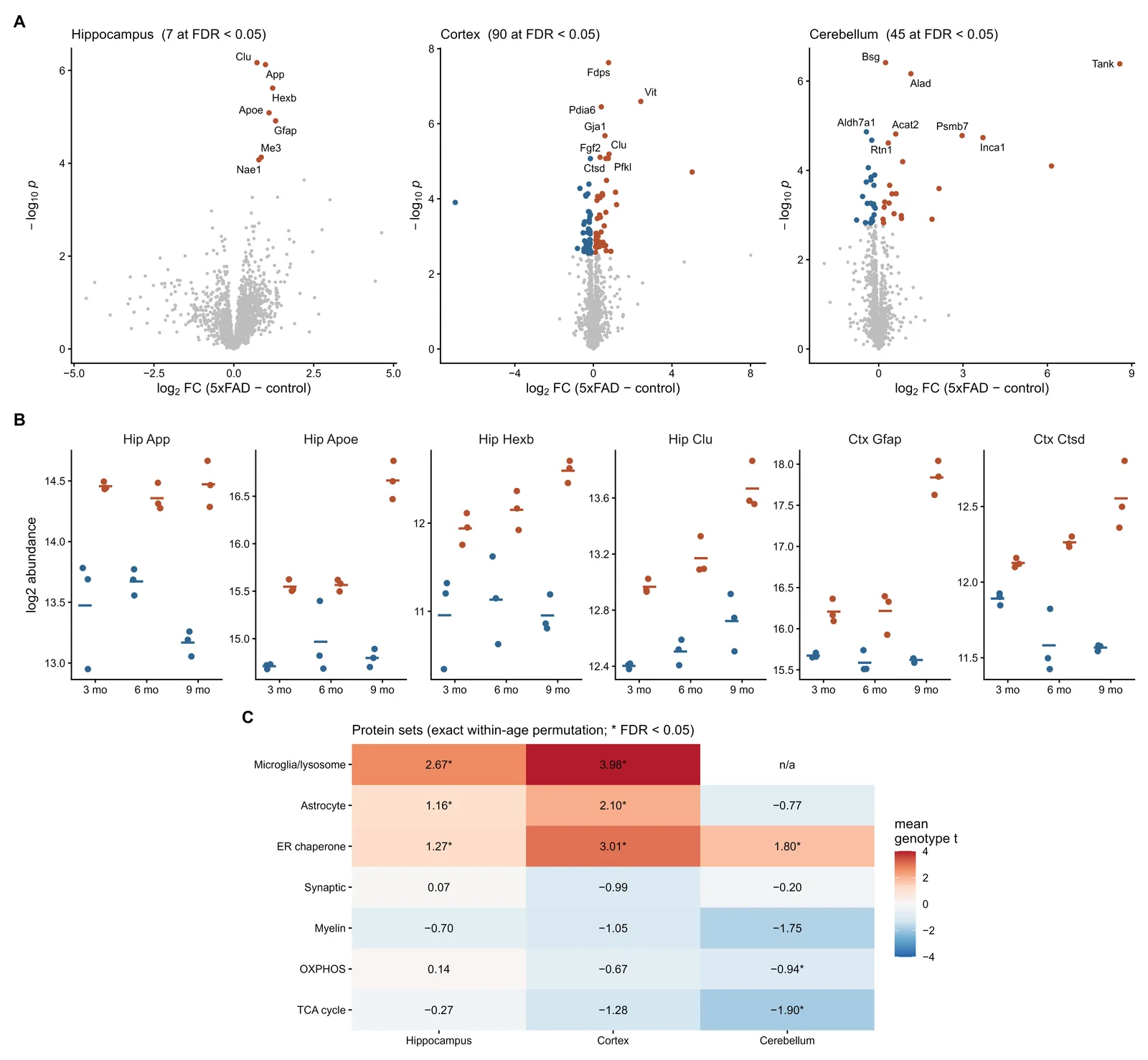
The 5xFAD brain proteome phenotype. (**A**) Age-adjusted genotype effects in hippocampus, cortex and cerebellum; red, higher in 5xFAD; blue, lower; gray, FDR ≥ 0.05. (**B**) Per-animal log2 abundances of representative proteins; blue, control; red, 5xFAD; horizontal bars, cell means; *n* = 3 per genotype and age. (**C**) Mean age-adjusted genotype t of prespecified protein sets, with significance from exact within-age permutation of genotype labels (8000 assignments) and Benjamini–Hochberg control across sets and regions; asterisks mark false discovery rate (FDR) < 0.05; n/a indicates fewer than three quantified members. FC, fold change; color scale, mean genotype t (red, higher in 5xFAD; blue, lower).

Age-adjusted moderated linear models identified seven proteins in the hippocampus that differed by genotype at a false discovery rate (FDR) below 0.05, all of them higher in 5xFAD animals (Figure 2A; Table S1). These were Clu (log2 fold change +0.72, FDR 7.6 × 10^−4^), App (+0.99, FDR 7.6 × 10^−4^), Hexb (+1.22, FDR 1.6 × 10^−3^), Apoe (+1.10, FDR 4.2 × 10^−3^), Gfap (+1.31, FDR 5.0 × 10^−3^), Me3 (+0.86, FDR 0.024) and Nae1 (+0.78, FDR 0.024). For App, Apoe, Clu and Hexb every transgenic value exceeded every control value, and the separations were already present at 3 months and largest at 9 months (Figure 2B). This recapitulates the signature reported by deep proteomic surveys of 5xFAD hippocampus and of human AD brain [20,21], and is concordant with our earlier proteomic studies of this model [22,23,24], which allows the present dataset to be anchored to an external reference before any integrative claim is made.

The cortex yielded 90 differentially abundant proteins of 1597 tested, 44 of them increased, including Fdps (+0.77, FDR 3.8 × 10^−5^), Pdia6 (+0.40, FDR 1.9 × 10^−4^), Gja1 (+0.59, FDR 8.3 × 10^−4^), Clu (+0.80, FDR 1.5 × 10^−3^), Ctsd (+0.63, FDR 1.5 × 10^−3^), Gfap (+1.13, FDR 6.7 × 10^−3^) and Cst3 (+0.47, FDR 6.7 × 10^−3^). Cortical App did not differ between genotypes (+0.11, FDR 0.84). Because App was quantified from peptides assigned to the mouse App sequence, App abundance in this dataset does not directly index expression of the human *APP* transgene in either region. The cerebellum yielded 45 differentially abundant proteins of 1473, including Apoe (+0.85, FDR 9.4 × 10^−3^) and Gfap (+0.80, FDR 0.045); one cerebellar protein with a very large fold change, Tank (+8.58), was detected in seven of nine 5xFAD and one of nine control animals, so its estimate reflects detection rather than abundance and it is not interpreted. The remaining large changes (S100a3 (+6.15), Inca1 (+3.71), Psmb7 (+2.97), Pip4k2b (+2.15)) were quantified in all 18 animals; they are reported in Table S1 and, because none belongs to a prespecified set and none has an established role in this model, they are not discussed further.

Because individual proteins are a weak basis for process-level statements at this sample size, we tested prespecified protein sets, defined from protein identity before any statistical comparison, using the mean age-adjusted genotype t of their quantified members against the exact null obtained by enumerating all 8000 within-age genotype label assignments (Figure 2C). Microglial and lysosomal proteins were coordinately elevated in the hippocampus (mean t 2.67, FDR 0.001) and cortex (3.98, FDR 0.001), and astrocytic proteins likewise in the hippocampus (1.16, FDR 0.029) and cortex (2.10, FDR 0.001). This pattern is the bulk-tissue counterpart of the plaque-associated, lysosomally reprogrammed microglial state described at single-cell resolution in this and related models [25]. Endoplasmic reticulum chaperone proteins were elevated in all three regions (FDR ≤ 0.008), consistent with the chaperone induction observed in human AD brain [26], although chaperone abundance alone does not demonstrate that the unfolded protein response is engaged.

Equally informative is where no difference was detectable. Synaptic, myelin, oxidative phosphorylation and tricarboxylic acid cycle protein sets showed no statistically detectable genotype association in the hippocampus or cortex, and only modest reductions in oxidative phosphorylation (mean t −0.94, FDR 0.042) and tricarboxylic acid cycle proteins (−1.90, FDR 0.011) in cerebellum. At these ages, and with this depth of proteome coverage, the 5xFAD hippocampal and cortical phenotype is therefore a glial and proteostatic one rather than a bioenergetic or synaptic one, and interpretations that invoke mitochondrial failure or synapse loss at 3 to 9 months are not supported by these data.

Genotype effects on cortical Gfap (interaction FDR 4.2 × 10^−4^) and Clu (interaction FDR 2.4 × 10^−3^) increased with age, with hippocampal Gfap and Apoe following the same trend at nominal significance. Aβ was not quantified, because bottom-up proteomics cannot distinguish Aβ from full-length APP. The amyloid-associated proteins that were quantified, App, Apoe, Clu and Hexb, separated the genotypes from 3 months, with the largest separation at 9 months (Figure 2B), consistent with the progressive Aβ accumulation and plaque deposition documented in this line [27,28]. Age itself was associated with 799 of 1597 cortical proteins but with no hippocampal and only one cerebellar protein. This regional asymmetry reflected a shift at 9 months, reaching several log2 units for individual proteins, that was of similar magnitude in both genotypes (Figure S3). Because all cortical samples were prepared together and acquired within one sequence in interleaved order, the shift is unlikely to reflect analytical batch, and its origin could not be determined. Because it affected both genotypes to a similar extent, it is captured by the age term of the model and does not account for the genotype or genotype-by-age effects reported here.

### 2.3. The Intestinal and Fecal Phenotype Is Dominated by Loss of Bile-Acid-Modifying Taxa

**Figure 3:**
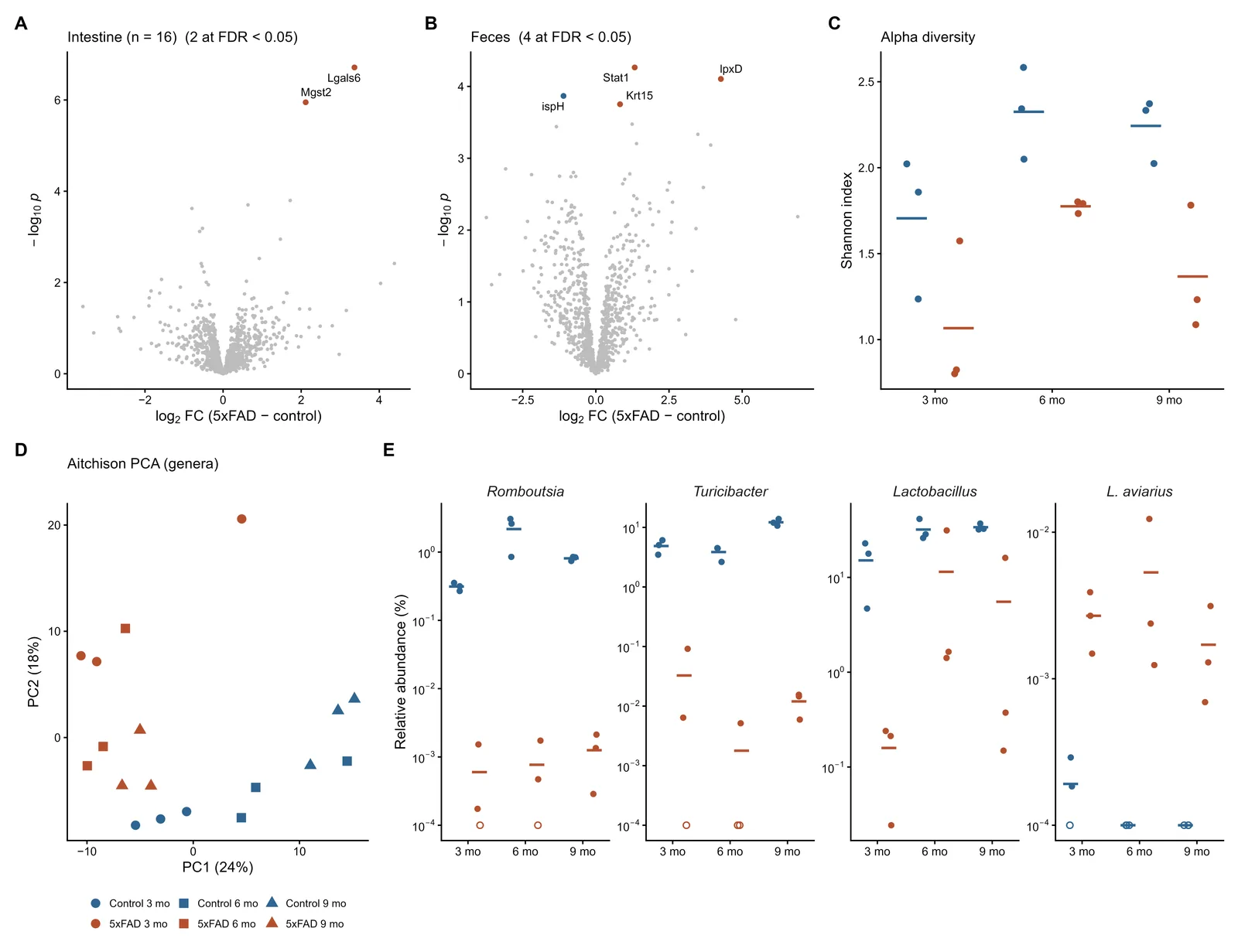
Intestinal, fecal and microbiome phenotypes. (**A**) Intestinal proteome (*n* = 16) and (**B**) fecal proteome, age-adjusted genotype effects. (**C**) Shannon diversity per animal. (**D**) Principal component analysis of genus-level centered log-ratio profiles in Aitchison geometry. (**E**) Relative abundance of *Romboutsia*, *Turicibacter*, *Lactobacillus* and *Ligilactobacillus aviarius* on a logarithmic scale; zero values are plotted at 10^−4^% as open circles. Blue, control; red, 5xFAD; horizontal bars, cell means of the plotted values; *n* = 3 per genotype and age. Genus composition of each animal is shown in Figure S4 and per-animal gut proteins in Figure S5. FC, fold change.

The intestinal and fecal proteomes separated by genotype as multivariate profiles but contained few individually significant proteins: Lgals6 (+3.36, FDR 2.1 × 10^−4^) and Mgst2 (+2.12, FDR 6.2 × 10^−4^) in intestine, and Stat1, LpxD, IspH and Krt15 in feces at FDR between 0.039 and 0.044 (Figure 3A,B and Figure S5). The fecal proteome mixes host and bacterial proteins, and differences in bacterial proteins track composition rather than activity; host-centric fecal proteomics has been proposed precisely because the host fraction reports on intestinal responses to the microbiota [29], and the present fecal proteome was not fractionated in that way.

Fecal microbial community structure separated the genotypes unambiguously. Shannon diversity was lower in 5xFAD animals at every age, with cell means of 1.07 against 1.71 at 3 months, 1.78 against 2.33 at 6 months and 1.37 against 2.24 at 9 months, and an exact within-age permutation *p* of 0.0015 (Figure 3C; Table S4). Species richness did not differ (*p* = 0.27), and the exact permutation test of multivariate dispersion, which measures the distance of each animal from its genotype × age centroid, detected no genotype difference (*p* = 0.22). Sequencing depth was, however, higher in 5xFAD animals (mean 24.5 against 17.3 million read pairs; *p* = 0.010), so each community-level measure was refitted by regression with depth as an additional covariate (Table S4). The lower Shannon diversity of 5xFAD animals persisted (−0.538 against −0.688 natural-log units without adjustment; *p* = 0.010), as did the lower Simpson index. Richness was strongly depth dependent (r = 0.87), and after adjustment it was lower in 5xFAD animals (*p* = 0.020), so the deeper sequencing of 5xFAD libraries may have masked a reduction in the number of detected taxa. In the same regression framework, distance to the genotype × age centroid was greater in 5xFAD animals after depth adjustment (*p* = 0.0012). This analysis differs from the permutation test: it treats the observed distances as fixed responses and relies on normal-theory inference, whereas the permutation test recomputes centroids and distances under every within-age genotype assignment; moreover, depth and genotype cannot be fully separated in this design. We therefore regard the depth-adjusted result as an exploratory sensitivity finding that neither replaces nor invalidates the permutation test. The lower diversity of 5xFAD communities is most securely attributed to reduced evenness, but contributions from lower richness and from greater within-group heterogeneity cannot be excluded. The depleted genera are present in control animals at percent-level abundance, several orders of magnitude above the detection limit at the lowest depth observed, so depth cannot account for their absence in 5xFAD animals, whose libraries were on average the more deeply sequenced. Community composition separated by genotype in Aitchison geometry with an exact within-age permutational analysis of variance *p* of 0.001, the smallest value attainable under this stratified enumeration (Figure 3D).

Twelve of 98 genera differed at FDR below 0.05. The dominant feature was the near-complete absence of *Romboutsia* and *Turicibacter* in transgenic animals: both genera were below 0.1% relative abundance in all nine 5xFAD mice at all three ages, against 0.27% to 13.9% in all nine controls (Figure 3E). *Lactobacillus* was reduced in 5xFAD animals (difference in centered log-ratio abundance, ΔCLR −3.39, FDR 1.1 × 10^−3^). At species level, 35 of 200 species differed at FDR below 0.05; among them, *Lactobacillus johnsonii* was reduced (ΔCLR −2.99, FDR 1.4 × 10^−3^) and *Ligilactobacillus aviarius* was higher in 5xFAD animals (ΔCLR +4.01, FDR 1.3 × 10^−6^) but present only at trace abundance (at most 0.012%; detected in all nine 5xFAD animals and in two of nine controls). Genotype effects varied with age for 26 genera.

Two features of this result deserve emphasis because they point in opposite interpretive directions. On the one hand, the taxa involved are not arbitrary with respect to the gut–brain literature. *Turicibacter* strains carry bile salt hydrolases and hydroxysteroid dehydrogenases that deconjugate and transform host bile acids, and colonization alters host bile acid profiles and systemic lipid handling [30]; *Turicibacter* abundance co-maps genetically with plasma cholic acid through the ileal bile acid transporter *Slc10a2* [31]; and the genus senses host serotonin through a neurotransmitter sodium symporter homolog, giving it a direct interface with host signaling [32]. Fecal proton nuclear magnetic resonance (1H-NMR) metabolomic profiling of the same animals, reported separately, identified bile-acid-related metabolites among the discriminating features at 6 and 9 months [12]. A depletion of bile-acid-modifying taxa is therefore a mechanistically tractable hypothesis rather than an isolated compositional observation.

On the other hand, the form of the difference is the form that housing effects take. The separation is absolute, present in every animal, and unchanged from 3 to 9 months, whereas a phenotype secondary to progressive amyloid pathology would be expected to emerge or deepen with age. In co-housed 5xFAD mice, *Turicibacter* differences appeared only at 18 months, and cage of origin explained more than 50% of microbiome variance in every comparison [11]; related models show cage contributions above 40% and taxon associations that differ in direction between lines [33,34]. Cage, litter and colony structure are among the strongest determinants of murine microbiota composition [35,36], and in this cohort littermates were separated by genotype at weaning, so that litter and maternal inoculum are balanced across genotypes, but cage is nested within genotype. Each analyzed animal, however, came from a different cage, so that every genotype and age group was represented by three cages; the near-complete absence of both genera in every transgenic animal is therefore replicated across nine independent cages rather than confined to one. We therefore describe these taxa as genotype-associated in this cohort and do not attribute them to amyloid pathology. Relative abundances describe proportions rather than absolute microbial loads [37]. Fecal microbial load can differ up to ten-fold between individuals [38], which could alter the magnitude of the genus-level differences reported here but is unlikely to account for the near-absence of *Romboutsia* and *Turicibacter* in every transgenic animal.

### 2.4. Integrated Six-View Analysis Identifies a Genotype-Associated Latent Structure

**Figure 4:**
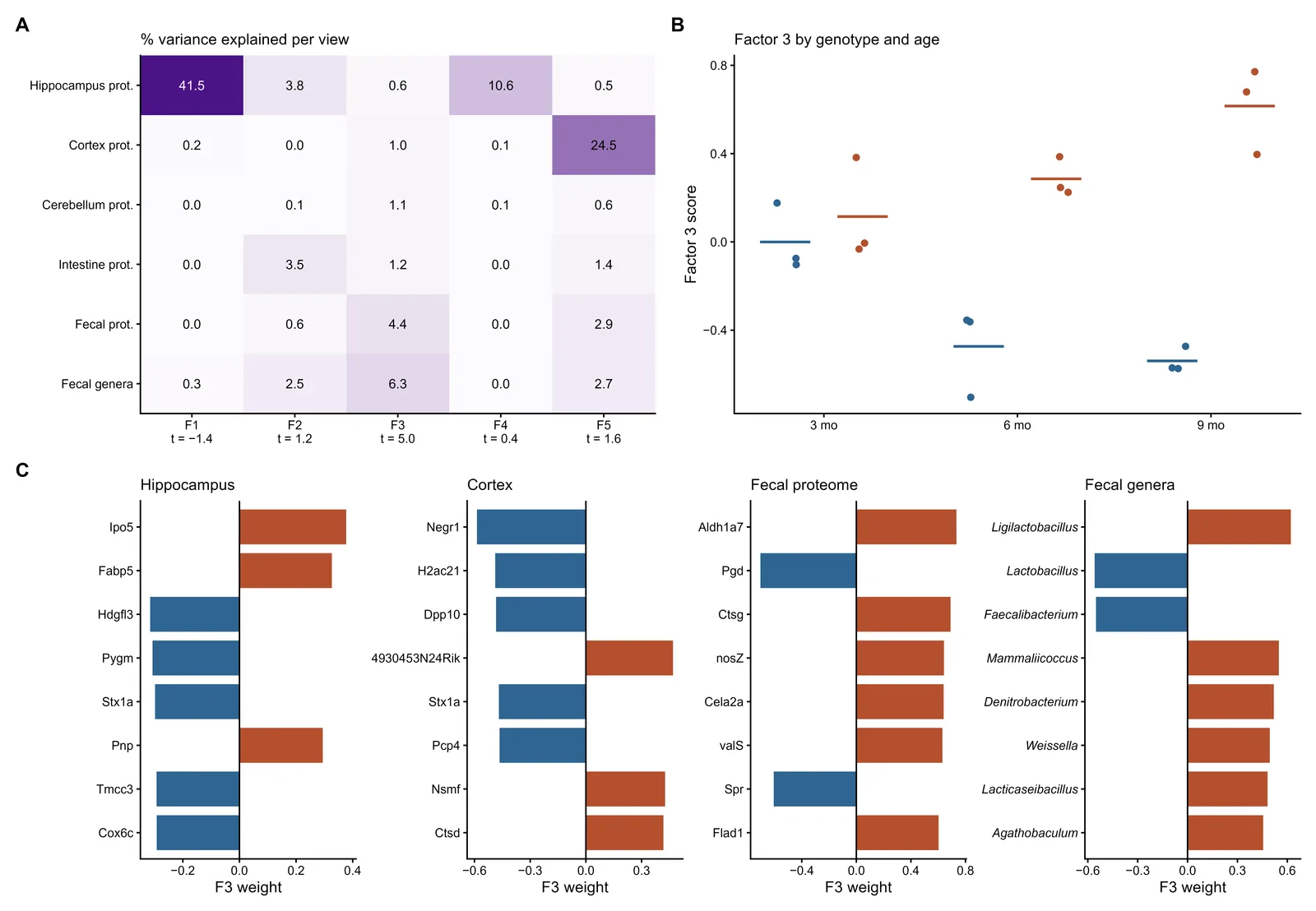
Shared latent structure across brain and gut compartments. (**A**) Percentage of variance explained by each multi-omics factor analysis (MOFA+) factor in each view; t values give the age-adjusted genotype coefficient of the factor scores; darker shading indicates more variance explained (**B**) Factor 3 scores by genotype and age. “blue, control; red, 5xFAD; horizontal bars, cell means”. (**C**) The eight largest Factor 3 weights in four representative views; red, positive; blue, negative. 4930453N24Rik, uncharacterized protein C3orf38 homolog (UniProt Q3TTL0).

MOFA+ applied to the six complete views retained five factors (Figure 4A; Table S5). Most of the variance in the model was not genotype related. Factor 1 accounted for 41.5% of hippocampal proteome variance and correlated with the number of features detected per sample (r = −0.64), identifying it as the sample-level intensity structure noted in Section 2.1; Factor 5 accounted for 24.5% of cortical variance and corresponded to the 9-month cortical shift (age F = 118.5, *p* = 1.7 × 10^−9^); Factor 4 captured a further 10.6% of hippocampal variance without genotype or age association. That the model isolates these sources into dedicated factors is the expected and desirable behavior of an unsupervised decomposition, and it is the reason a genotype-associated factor can be read as something other than residual technical structure.

**Figure 5:**
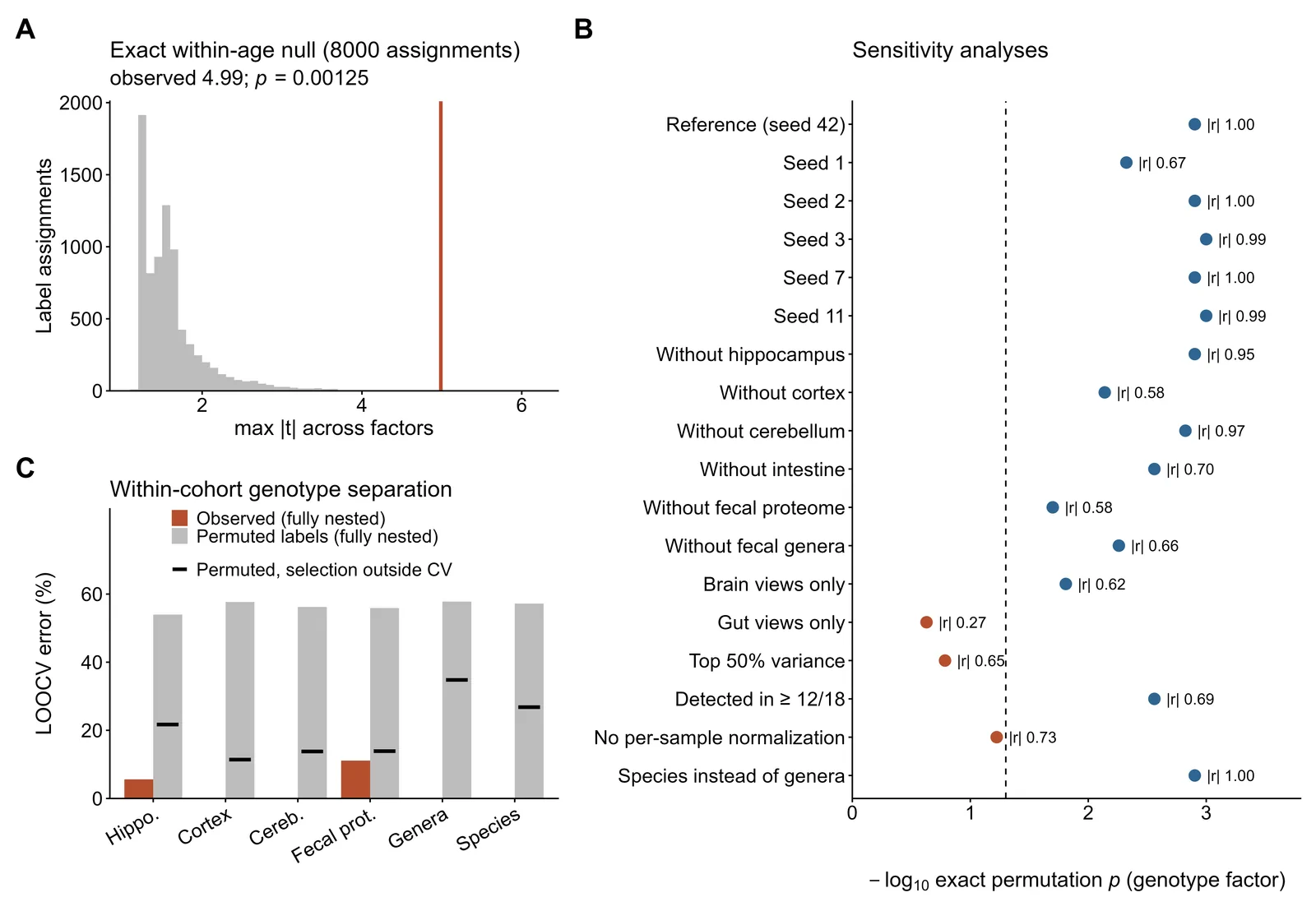
Permutation calibration, robustness and within-cohort separation. (**A**) Exact null distribution of the maximum absolute genotype t across factors over all 8000 within-age genotype assignments, with the observed value marked. “red line, observed value”. (**B**) Exact permutation *p* values for the genotype-associated factor across initializations, view omissions and preprocessing variants; |r| gives the correlation of each recovered factor with the reference factor; “blue, *p* < 0.05; red, *p* ≥ 0.05;” dashed line, *p* = 0.05. (**C**) Leave-one-out cross-validation (LOOCV) classification error (red bars) with imputation, feature selection and scaling all performed inside each training fold, compared with permuted-label performance (gray bars;999 within-age permutations); black ticks show permuted-label error when feature selection is instead performed once on all animals.

Factor 3 was associated with genotype after adjustment for age (t = 4.99). Calibrated against the exact distribution of the maximum absolute genotype t across all factors over all 8000 within-age label assignments, which controls the family-wise error rate across factors by construction, this corresponds to *p* = 0.00125 (Figure 5A). The factor spanned compartments, explaining 6.3% of fecal genus variance, 4.4% of fecal proteome variance, and between 0.6% and 1.2% of variance in each brain and intestinal proteome (Figure 4A). Its weights were carried disproportionately by the microbial phenotype: *Ligilactobacillus*, *Lactobacillus* and *Faecalibacterium* held three of the four largest genus weights and *Romboutsia* the ninth of 98, whereas the canonical amyloid-associated brain proteins ranked between 114th and 330th of 2,032 in the hippocampal view (Figure 4C). The largest brain weights were instead carried mainly by proteins outside the canonical amyloid-associated set; the lysosomal protease Ctsd, a plaque-associated microglial protein, ranked eighth of 1,597 in the cortical view. The shared axis is therefore not the brain amyloid signature propagating into the gut views. The 5xFAD—control difference in mean Factor 3 score was 0.12 at 3 months, 0.76 at 6 months and 1.16 at 9 months (genotype × age interaction, parametric F = 14.9, *p* = 5.6 × 10^−4^; Figure 4B). The increase reflected both a decline in control scores after 3 months and a progressive rise in 5xFAD scores, and with three animals per cell this age pattern is reported descriptively.

Two properties of this factor limit how far it can be pushed. The first is its size: a component explaining at most 6.3% of variance in any view describes a real but minor axis of the dataset, not its dominant organization. The second is that the proteins driving the brain phenotype in Section 2.2 are not the proteins driving this factor, so the shared structure and the amyloid signature are partly distinct features of the same dataset.

### 2.5. Robustness, Exact Permutation Inference and Within-Cohort Separation

Because latent factors recovered from 18 animals and several thousand features can be fragile, we assessed the genotype-associated factor across initializations, view composition and preprocessing choices (Figure 5B). It was recovered in the reference fit and in all five additional random initializations, with exact permutation *p* between 0.001 and 0.005; four of the five additional runs correlated with the reference at |r| ≥ 0.99, and one yielded a compositionally different solution at |r| = 0.67. Omitting any single view left a genotype-associated factor with *p* between 0.001 and 0.020, but its composition shifted appreciably in several cases, with |r| against the reference ranging from 0.58 to 0.97. A model restricted to the three brain proteomes gave a weaker association (*p* = 0.016) and a model restricted to the gut views gave none (*p* = 0.24); the brain-only factor was, moreover, compositionally different from the reference (|r| = 0.62), so the six-view factor is not reproduced by either compartment alone. The association persisted under a stricter detection filter (*p* = 0.003, |r| = 0.69) and when species replaced genera (*p* = 0.001), but was lost when features were restricted to the most variable half of each view (*p* = 0.16) and when per-sample normalization was omitted (*p* = 0.06). The shared genotype-associated structure is therefore present under the preprocessing specified in advance but is not robust to every reasonable alternative.

Within-cohort genotype separability was high in individual views. Leave-one-out classification with feature selection repeated inside every training fold gave errors of 5.6% for the hippocampal proteome, 0% for the cortical and cerebellar proteomes, 0% for fecal genera and species, and 11.1% for the fecal proteome, against mean errors between 54% and 58% for permuted labels, with empirical *p* values between 0.001 and 0.006 in all views (999 within-age permutations; Figure 5C). These figures describe separation within this cohort and are not estimates of diagnostic performance. Their interpretation depends entirely on nesting: when feature selection was instead performed once on all animals before cross-validation, permuted labels still produced mean errors of only 11% to 35%, reproducing the selection bias documented for high-dimensional classifiers [18,19] and illustrating how readily a design of this size generates apparently decisive but uninformative accuracies.

### 2.6. Individual-Level Gut–Brain Coupling Is Not Detectable in This Design

The analyses above establish that genotype is associated with molecular differences in both compartments and that those differences share a latent axis. Neither result addresses whether an individual animal’s gut state predicts its brain state. To ask that question we removed all group structure by centering every feature within its genotype and age cell, and then computed correlations between brain proteins, of which 4709 were complete, and the 98 fecal genera, summarizing the result as the proportion of the 461,482 pairs reaching nominal *p* < 0.05. The null was constructed by permuting animal identities within cells in one view, which destroys within-animal linkage while preserving group membership exactly.

**Figure 6:**
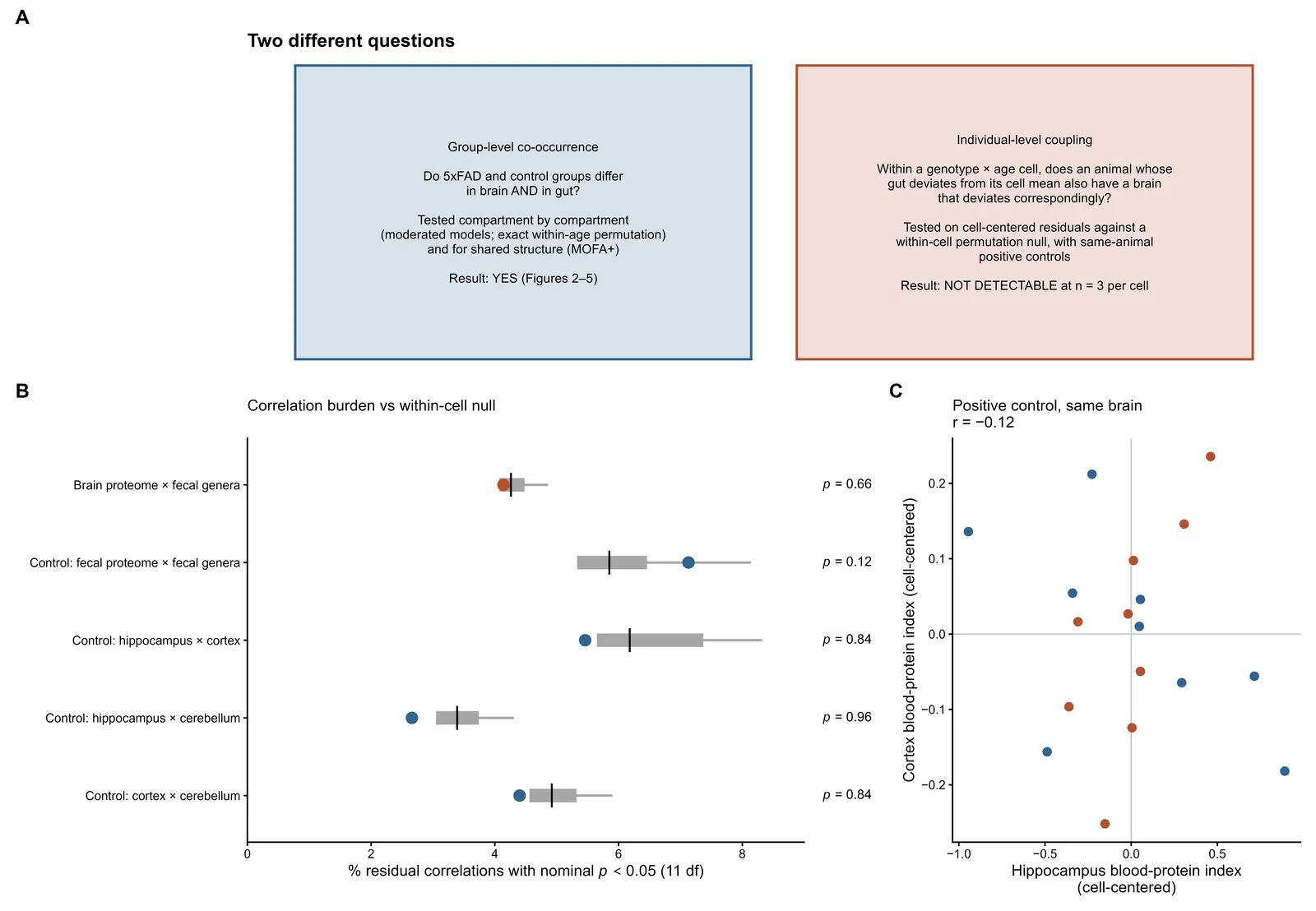
Group-level co-occurrence and individual-level coupling are different questions. (**A**) The two questions, the test applied to each and the outcome. (**B**) Percentage of cross-view residual correlations reaching nominal *p* < 0.05 at 11 residual degrees of freedom (points; red, brain–gut comparison; blue, positive controls) against the within-cell permutation null from exact enumeration of all 46,656 within-cell permutations (gray bars, interquartile range; thin lines, extension to the 95th percentile; black ticks, median), for the brain–gut comparison and four same-animal positive controls. (**C**) Cell-centered blood-protein index in hippocampus against cortex of the same animal; blue, control; red, 5xFAD.

Observed correlation burden was 4.14%, against a null median of 4.26% (95th percentile 4.86%), giving an exact *p* of 0.66 (Figure 6B). There was thus no evidence of excess individual-level brain–gut covariation.

This negative result is interpretable only against positive controls (Figure 6A,B). Pairs of measurements that must covary within an animal if the design can detect covariation at all were likewise null: fecal proteome against fecal genera, derived from the same material, gave *p* = 0.12; hippocampus against cortex gave *p* = 0.84; hippocampus against cerebellum *p* = 0.96; and cortex against cerebellum *p* = 0.84. An index of blood-derived proteins, which should track perfusion and dissection differences common to all regions of a single brain and therefore offers about as favorable a test of within-animal linkage as this dataset allows, did not correlate across regions within cells either (r between −0.12 and 0.22; Figure 6C). A taxon-specific pair, *Clostridioides difficile toxin A* (TcdA) in the fecal proteome against *C. difficile* abundance in the metagenome of the same pellet, was likewise uncorrelated within cells (r = −0.16; exact *p* = 0.59), although toxin production is regulated and need not track abundance. Because each tissue was acquired in a separate sequence, technical variation is independent across brain regions, so these between-region controls test shared biological rather than shared analytical variation. After the six genotype-by-age cell means are removed, eleven degrees of freedom remain for each residual correlation, and the total information available for within-animal covariation is correspondingly small relative to the sample-level variation of each assay. The correct conclusion is not that the gut and brain are uncoupled in 5xFAD mice, but that this design, which is typical in scale of the observational literature on which gut–brain inferences in AD models are routinely based, can exclude coupling that is strong and moderately broad, or weak but dense, but not coupling that is sparse or weak. Power depended jointly on the strength of coupling and on the proportion of features involved (Table S6d): at ρ ≥ 0.7, power was at least 90% when 10% or more of the features in both compartments were coupled, and at ρ = 0.5 when 25% or more were coupled; at ρ = 0.3, power was 0% when 5% and 0% when 10% of features were coupled, 18% at 25% and 100% at 50%; when only 5% of features were coupled, power was below 10% at every correlation tested. The positive controls convert an otherwise uninterpretable null into a quantitative statement about design adequacy. Because the same-animal positive controls were also null, this result is not evidence against individual-level coupling. It shows that within-animal brain–gut covariation, if present, is sparse or weak relative to the residual variation of each assay; with 11 residual degrees of freedom, an observed correlation of 0.5 has a 95% confidence interval of about −0.07 to 0.82, and correlation estimates generally stabilize only at several hundred observations [39]; a non-significant result at this size is therefore absence of evidence rather than evidence of absence [40].

### 2.7. Exploratory Untargeted LC-MS Metabolomic and Lipidomic Profiles

Untargeted metabolomic and lipidomic profiles were acquired for all five sample types (ten matrices; Section 4.5) and were exported separately for each age after filtering for genotype differences (ANOVA *p* < 0.05 and fold change ≥ 1.5). Consistent with this selection, 99.8% of the 5,659 feature-by-age values differed between genotypes at nominal *p* < 0.05 and 96.1% by at least 1.5-fold, and only 0–6.3% of features per matrix were retained at all three ages (Figure S1). Because these feature lists were selected on the genotype contrast and largely do not overlap across ages, they cannot support unbiased genotype inference or integration across ages; in an exploratory model that included them, the age pattern of the dominant LC-MS-derived factor depended on initialization (Figure S6). These matrices were therefore excluded from the differential and integrative analyses and are reported descriptively, with the annotated feature lists provided in Table S3.

A separate and more informative metabolic line of evidence exists for these animals. Fecal ^1^H-NMR metabolomic profiling of these animals identified cholate among the discriminating metabolites at 6 and 9 months, alongside trimethylamine N-oxide, phenylacetate and glucose at 6 months [12]. That analysis is published separately and is not repeated or re-analyzed here; its cholate observation is therefore a concurrent finding in the same animals rather than a result of the present study. Read alongside the depletion of bile-acid-modifying taxa reported in Section 2.3, it constitutes the most direct hypothesis this dataset generates, and Section 3 treats it as such.

### 2.8. Summary of the Principal Findings

Table 2 collects the findings that survive the analyses above, together with the level of evidence each is entitled to. The distinction matters because the same dataset supports direct observation in the brain, statistical inference with an unresolved confounder in the microbiome, and, for the shared latent axis, an inference about covariance that is explicitly not an inference about communication.

## 3. Discussion

The central result of this study is a dissociation. In a single cohort of 5xFAD mice, genotype was associated with molecular differences in every compartment examined, from hippocampus to fecal microbiota, and those differences shared a latent axis that survived exact permutation testing. Yet when the same animals were interrogated for individual-level covariation between gut and brain, no signal exceeded the null, and the same was true of positive controls that must covary if the design can detect covariation at all. The two halves of this result correspond to the two claims that the gut–brain literature in AD tends to merge, and separating them changes what the data can be said to show.

The brain phenotype is the most secure part of the dataset and serves as its internal reference. The elevation in App, Apoe, Clu, Hexb and Gfap reproduces deep proteomic characterizations of 5xFAD hippocampus and of human AD brain [20,21] and our own prior measurements in this model [22,23,24], and the presence of these differences at 3 months is consistent with amyloid deposition from approximately 2 months of age in this line [27]. The set-level analysis adds something the individual proteins do not. Coordinated elevation in microglial, lysosomal and astrocytic proteins, without statistically detectable change in synaptic, oxidative phosphorylation or tricarboxylic acid cycle sets in the hippocampus or cortex, places this cohort at a stage in which glial reprogramming is established but bulk bioenergetic and synaptic protein complements are not yet measurably altered. This is the tissue-level counterpart of the disease-associated microglial state characterized transcriptionally in the same model [25], and it usefully constrains interpretation: at 3 to 9 months, a mitochondrial or synaptic narrative would not be supported by these proteomes. The elevation in endoplasmic reticulum chaperones across regions is consistent with the chaperone induction reported in AD brain [26] but does not by itself establish unfolded protein response signaling, which would require phosphorylation-state measurements. The increases in cerebellar Apoe and Gfap are notable because plaques are sparse in 5xFAD cerebellum [27], and we did not determine their basis.

The gut phenotype is the most interesting and the least resolved. Two considerations pull against each other. The taxa that distinguish the genotypes are precisely those with a characterized interface to host bile acid and monoamine metabolism: *Turicibacter* strains modify conjugated bile acids through strain-specific bile salt hydrolases and alter host lipid profiles on colonization [30], their abundance co-maps genetically with plasma cholic acid via the ileal transporter *Slc10a2* [31], and they import host serotonin through a dedicated transporter homolog [32]. Fecal ^1^H-NMR profiling of the same animals placed cholate among the discriminating features at 6 and 9 months [12], so the compositional and metabolic observations converge on the same axis. This axis is increasingly well defined: host metabolism actively counterbalances microbial bile acid transformation through re-conjugation and synthesis [41], and circulating bile acid profiles, in particular reduced cholic acid and elevated secondary bile acids, have been associated with dementia and its progression in human cohorts [42]. In 5xFAD mice, a dietary intervention that remodeled the microbiota and lowered circulating bile acids was accompanied by reduced hippocampal amyloid and microglial activation [43], and in APP/PS1 mice mediation analysis placed fecal deoxycholic acid between a gut genus and disease status [44], although in that model *Turicibacter* was enriched rather than depleted. Notably, *Turicibacter* and *Romboutsia* *ilealis*, as well as *Lactobacillus johnsonii* were also among the taxa depleted in 5xFAD relative to wild-type littermates in an independent colony [11], so the taxonomic identity of the depletion replicates across facilities even though its timing does not. Against this, the pattern in our data is complete at 3 months and unchanged thereafter, which is the signature of a fixed environmental difference rather than of a phenotype secondary to progressive pathology, and which differs from the longitudinal course reported in co-housed 5xFAD mice, where *Turicibacter* changes appeared only at 18 months and cage explained most of the variance [11]. Because cage is nested within genotype after weaning, the appropriate description is that these taxa are genotype-associated in this cohort, that the association is replicated across cages but cannot be fully separated from genotype-specific housing, and that the bile acid link is a hypothesis for a cage-controlled experiment rather than a conclusion. The mechanistic hypothesis is nevertheless testable, and the alternative interpretations are distinguishable by design rather than by reanalysis.

The integrative result requires the most careful phrasing. A genotype-associated factor spanning brain, intestinal and fecal views demonstrate that, across animals and without conditioning on genotype, deviations in these compartments are not independent, and that a single low-dimensional summary captures part of what genotype does in both. It does not demonstrate transfer of information between compartments, and it could not: any set of views that each differ by genotype will tend to share a genotype-aligned component. The model’s own variance decomposition reinforces the point, since the genotype factor is minor relative to sample-level and age-associated factors, and the canonical amyloid proteins are absent from its top brain weights. The factor is also preprocessing-dependent, disappearing under a variance filter and without per-sample normalization. We regard reporting this dependence as part of the result rather than as a concession, because a latent factor that is reported without sensitivity analysis cannot be distinguished by a reader from one that would not survive it.

Because the factor is minor in magnitude and its weights are carried mainly by the microbial phenotype rather than by the canonical amyloid proteins (Section 2.4), the shared structure and the brain amyloid signature are partly distinct features of the same dataset, which is itself informative: the compartments are linked in the model by something other than a simple propagation of the amyloid phenotype. What that something is cannot be determined from these data. It is appropriately described as a genotype-associated cross-view latent structure of modest magnitude, and not as a dominant, global or organism-wide axis, still less as a signaling pathway.

The individual-level analysis is where this study departs from the observational literature it draws on. Interventional work has established that the microbiota can modify amyloid pathology when perturbed, whether by germ-free rearing [3], microbiota transfer [4], probiotic or prebiotic treatment [5,7] or antibiotic exposure [6]. Observational work has established that gut composition, gut barrier proteins and gut metabolites differ between transgenic and wild-type animals [9,10,11,12,13,14]. The step that is usually assumed rather than tested is that these compartment-level differences are connected within individuals in an unperturbed cohort. Our data indicate that in a design of this scale only broad, strong coupling can be excluded, and the positive controls make that statement quantitative: measurements taken from the same brain, or from the same fecal sample, showed no more within-animal covariation than permuted data. We would emphasize the implication for study design rather than for gut–brain biology. Detecting individual-level coupling requires either substantially more animals per cell, replicate measurements that allow assay-level noise to be separated from biological variation, or designs in which coupling is manipulated rather than observed. Reporting a null without controls of this kind, in a cohort of this size, would not have been informative in either direction.

We regard this as the most useful part of the study, because the distinction it enforces is one the field does not usually make. A great deal of observational work in AD models reports a microbiome difference and a brain difference in the same animals and treats their co-occurrence as evidence of an interacting axis. Co-occurrence at the group level, however, follows from any genotype that perturbs both compartments independently, and our data supply exactly that situation: every compartment differs by genotype, the differences share a latent structure, and yet nothing detectable in the individual-level structure connects them. Whether that reflects genuine independence or insufficient resolution is not answerable here, and the null positive controls indicate the latter is at least as likely. Either way, the group-level result cannot serve as evidence for the individual-level claim, and studies intended to support gut–brain inference should be sized and controlled for the question they are asking rather than for the question that is easier to answer.

Several limitations bound these conclusions. The cohort comprises 18 male animals with three per genotype and age cell and is cross-sectional, so no causal or mechanistic inference is possible and sex effects, which modify amyloid pathology in this model [28], cannot be assessed. Controls were littermates of the transgenic animals, which balances litter and maternal microbiota across genotypes, but separation by genotype at weaning nests cage within genotype, and despite replication across cages this is the principal unresolved biological confounder for the microbiome findings; this is a housing and environment issue rather than an analytical one, since all samples of each tissue were prepared together and acquired in a single sequence with genotype and age interleaved. Because fecal material was collected from each animal individually and sample-to-animal linkage was verified against laboratory records (Table S1), the individual-level analysis is limited by sample size rather than by pooling or linkage uncertainty. The untargeted LC-MS matrices as supplied were filtered on the genotype contrast and can serve only exploratory purposes, the classification results describe within-cohort separation and not generalizable performance, relative abundances constrain microbiome interpretation to proportions rather than loads, and the fecal ^1^H-NMR profiling of the same animals reported separately [12] is an overlapping dataset rather than independent replication.

Because only males were studied, the results cannot be generalized to females. Sex modifies amyloid pathology and other phenotypes in this model [28], so genotype effects on glial and lysosomal proteins may differ in magnitude in females. Sex and gonadal hormones also shape murine microbiota composition and bile acid profiles [45], which bears directly on the bile-acid-modifying taxa reported here. Antibiotic perturbation of the microbiota reduced cerebral amyloidosis and altered microglial phenotypes in male but not female APPPS1-21 mice [46]. Microbiota–amyloid coupling may therefore be sex-dependent, and neither the shared latent factor nor the bound on individual-level coupling reported here can be extrapolated to females.

Cage effects in laboratory mice develop through successional community assembly over several weeks and are not removed by a shared founding inoculum [47]. Separation at weaning therefore gives genotype-specific cage communities time to diverge, and the genus-level differences reported here may be partly or wholly housing effects. A shared cage environment acting on both compartments would create rather than mask within-cell brain–gut covariation, so the individual-level result is not explained by cage structure. Resolving the microbiome findings requires co-housing of genotypes, several cages per group with cage modeled as a random effect, or bedding transfer between cages [35,36].

Whether *Romboutsia* and *Turicibacter* are absent or diluted, and whether total microbial load differs by genotype, requires absolute quantification by flow cytometry, quantitative PCR or spike-in standards [38]. No extraction or library-preparation negative controls were sequenced, and the sequencing service record confirms that only the 18 fecal samples were processed in the run. Contaminant DNA therefore cannot be excluded directly, but its influence is expected to be small in high-biomass fecal material [48], the genotype-associated genera are abundant gut commensals rather than typical reagent contaminants, and the dominance of the environmental genus *Curtobacterium* in one sample (M03) did not affect the genotype-associated results: excluding this animal, no genus lost significance, *Romboutsia* and *Turicibacter* remained depleted (ΔCLR −7.34 and −8.71; FDR 6.1 × 10^−8^ and 1.0 × 10^−7^) and the genotype difference in Shannon diversity was unchanged in direction (t = −5.83 against −4.79 in the full cohort; Table S4). Finally, protein changes were quantified by label-free data-independent mass spectrometry and were not confirmed by antibody-based methods, although peptide-specific mass spectrometric quantification is itself an established alternative to immunoblotting [49]; the individual proteins discussed reproduce published 5xFAD proteomes [20,21,22,23,24], and process-level conclusions rest on protein-set tests rather than on single proteins.

Three follow-up experiments would resolve most of what is open here. A sex-balanced cohort in which littermates of both genotypes remain co-housed after weaning would determine whether the *Romboutsia* and *Turicibacter* depletion persists when the cage environment is shared. Targeted quantification of conjugated and unconjugated bile acids in intestinal content and serum, alongside absolute microbial load, would test the bile acid hypothesis directly rather than through relative abundances. And a perturbation design, whether gnotobiotic colonization with defined *Turicibacter* strains or fecal transfer between genotypes, would convert the covariance structure described here into a causal statement, which no amount of cross-sectional profiling can do.

## 4. Materials and Methods

### 4.1. Animals and Ethics

Male 5xFAD mice and controls were studied at 3, 6 and 9 months of age, with three animals per genotype at each age and different animals used at each age. Hemizygous 5xFAD mice (Tg6799; Mutant Mouse Resource and Research Centers (MMRRC) stock #034840-JAX), which carry five familial AD mutations in human *APP* and *PSEN1* transgenes, and B6SJLF1/J mice (stock #100012) were obtained from The Jackson Laboratory (Bar Harbor, ME, USA) and crossed to generate the study cohort. Offspring were genotyped by PCR on genomic DNA from ear biopsies following the Jackson Laboratory protocol with primers for the APP transgene (forward 5′-AGGACTGACCACTCGACCAG-3′, reverse 5′-CGGGGGTCTAGTTCTGCAT-3′; 377 bp), the PSEN1 transgene (forward 5′-AATAGAGAACGGCAGGAGCA-3′, reverse 5′-GCCATGAGGGCACTAATCAT-3′; 608 bp) and an internal control (forward 5′-CTAGGCCACAGAATTGAAAGATCT-3′, reverse 5′-GTAGGTGGAAATTCTAGCATCATCC-3′; 324 bp) (94 °C for 3 min; 35 cycles of 94 °C for 30 s, 57.3 °C for 60 s and 72 °C for 60 s; final extension at 72 °C for 2 min), with amplicons resolved on 3% agarose gels. Wild-type littermates served as controls. Littermates were separated by genotype at weaning and housed in genotype-specific social cages, with each genotype and age group distributed across three cages, in individually ventilated cages at 20 °C under a 12 h light/12 h dark cycle with food and water available ad libitum [12]. Each of the 18 analyzed animals came from a different cage, so that nine cages contributed 5xFAD animals and nine contributed controls, with one analyzed animal per cage. Because cage is nested within genotype, cage and genotype effects are aliased at the cage level and cannot be separated in these data, and because each cage contributed a single animal, cage and animal coincide and a separate cage random effect is not estimable. Cage was therefore not modeled, the animal, and thus the cage, was the experimental unit, and all microbiome results are reported as genotype-associated within this housing structure. Investigators were not blinded to genotype during sample collection or analysis; data processing and statistical analyses were performed with automated software pipelines. Sample size (*n* = 3 per genotype and age) was determined by the availability of age-matched littermates and is consistent with our previous proteomic studies in this model [22,23,24]; its implications for statistical power are addressed in Section 2.6. All procedures were approved by the Acibadem Mehmet Ali Aydinlar University Ethics Committee for Experimental Research on Animals (ACU-HADYEK, Istanbul, Türkiye; approval no. ACU-HADYEK 2022/71, 7 December 2022) and performed in accordance with institutional guidelines, and reporting follows the ARRIVE 2.0 guidelines [50].

### 4.2. Sample Collection and Analytical Workflow

At each scheduled age animals were anesthetized with isoflurane and euthanized by decapitation; the hippocampus, cortex, cerebellum and the distal colon were dissected and frozen, and fresh fecal pellets were collected individually from each animal: each mouse was placed alone in a clean, empty container without bedding, and pellets were collected with sterile forceps immediately after defecation, transferred to sterile tubes, frozen immediately on dry ice and stored at −80 °C until analysis. All collections were performed at the same time of day (12:00–14:00) to minimize diurnal variation in microbiota composition.

Analytical processing began only after tissues and fecal material had been collected from all 18 animals. All samples of a given tissue were then prepared together by filter-aided sample preparation (FASP) under a single standardized protocol. Each tissue was subsequently acquired as a separate LC-MS/MS sequence of approximately two days, in which the 18 samples of that tissue were run in an interleaved order in which the three ages and both genotypes were intermixed rather than grouped; samples of different tissues were not intermixed within a sequence, and each sequence was started only after the preceding one had been completed and instrument performance had been verified. Acquisition blocks therefore coincide with tissue and are orthogonal to genotype and age. Within all tissue, genotype and age comparisons were made among samples that had been prepared together and measured in the same sequence, so that age and genotype are biological rather than analytical variables in all models and no batch covariate or batch-correction procedure was required. Because each data view corresponds to a single acquisition block, differences in absolute intensity between tissues were not interpreted. The animal was the experimental unit throughout, and one measurement per animal per view entered the analysis. Each sample was traced to its animal identifier through laboratory records; the mapping of every raw file and sequencing sample to its animal, and the verification procedure, are given in Table S1.

### 4.3. Proteomics

Proteomic sample preparation, acquisition and data processing followed our published workflow [51,52]. Briefly, tissues were homogenized with steel beads, lysed in UPX buffer (Expedeon, Cambridge, UK) containing a protease inhibitor cocktail (Thermo Scientific, Waltham, MA, USA) by sonication, heated at 95 °C for 10 min and centrifuged at 14,000× *g* for 10 min. Up to 100 µg of protein was processed by filter-aided sample preparation (FASP; Expedeon) with washes in 8 M urea, alkylation with 50 mM iodoacetamide, buffer exchange to 25 mM ammonium bicarbonate and tryptic digestion (enzyme-to-protein ratio 1:100), and peptides were adjusted to 200 µg/mL in 0.1% formic acid. Peptides were separated on an ACQUITY UPLC M-Class system (Symmetry C18 trap column, 5 µm, 180 µm × 20 mm; CSH C18 analytical column, 1.7 µm, 75 µm × 250 mm) coupled to a Xevo G2-XS QTof mass spectrometer (Waters, Milford, MA, USA) operated in positive-ion SONAR data-independent acquisition mode (24 Da quadrupole transmission window, *m*/*z* 50–1950), with Glu-1-fibrinopeptide B (100 fmol/µL) as lock mass. Each sample was injected in technical duplicate and replicate runs were combined in Progenesis. Data were processed in Progenesis QI for proteomics (v4.1; Waters) against the reviewed UniProt *Mus musculus* database (2024 release), with carbamidomethylation of cysteine as a fixed modification, protein N-terminal acetylation, methionine oxidation and asparagine/glutamine deamidation as variable modifications, at most one missed cleavage, at least two fragment ions per peptide, five fragment ions per protein and at least one peptide per protein, a protein-level false discovery rate below 1%, and total ion intensity normalization. Identified proteins for all proteomes, with accession numbers and peptide counts, are listed in Data S1. Intestinal and fecal proteomes were processed with the same workflow; for the fecal proteome, protein sequences of bacterial families known to occur in the mouse gut were downloaded and combined with the mouse sequences into a single FASTA search database, following our metaproteomic workflow [13]. For the present analysis, proteins were collapsed to gene symbols retaining the entry with the greatest number of non-missing values, features detected in fewer than 6 of 18 animals were removed, intensities were log2-transformed after addition of half the smallest positive value in each view, and each sample was median-centered.

### 4.4. Shotgun Metagenomics

DNA extraction, library preparation and sequencing were performed as an outsourced service by SZA Omics (Istanbul, Türkiye). Total DNA was extracted from approximately 50 mg of fecal material per sample with the QIAsymphony PowerFecal Pro DNA Kit on the QIAsymphony SP automated platform (QIAGEN, Hilden, Germany). Shotgun metagenomic libraries were prepared from total DNA, without marker-gene amplification, with the Illumina DNA Prep (M) kit (formerly Nextera DNA Flex; Illumina, San Diego, CA, USA) using the standard-input, bead-linked transposome (tagmentation) workflow, and sequenced on an Illumina NovaSeq 6000 platform in paired-end 2 × 150 bp mode, yielding 13.8–34.0 million read pairs per sample (mean 20.9 million; 27.6–68.0 million individual reads). Eighteen fecal samples were submitted for sequencing and no extraction or library-preparation negative controls were included in the run. Per-sample DNA quality, library quality, index assignment, raw read counts (as read pairs and as individual reads), Q30 scores, the numbers of read pairs retained after length filtering, removed as host-derived and assigned at species level, and the classification and human-read proportions of the sequencing service’s own Kraken2 run are given in Table S4; the length-filtering and host-removal counts were re-derived from the delivered FASTQ files with the same software versions, parameters and reference as the original processing. Sequencing depth was not balanced across genotypes (5xFAD mean 24.5 million read pairs, range 13.8–34.0; controls mean 17.3 million, range 15.6–20.6; genotype effect +7.1 million read pairs, *p* = 0.010; the four most deeply sequenced libraries were all from 5xFAD animals and were sequenced on a separate lane of the same flow cell), so every community-level measure was additionally refitted by ordinary least squares with depth as a covariate (Table S4); these regressions are exploratory sensitivity analyses and do not replace the exact permutation tests. Reads were processed in QIIME 2 (version 2025.4) using the MOSHPIT metagenomics workflow [53]. Reads were length-filtered with Cutadapt (version 5.0) [54], discarding read pairs in which a read was shorter than 90 bp (no adapter or quality trimming parameters were set), and host-derived reads were removed by local-mode alignment with Bowtie2 (version 2.5.1; sensitive preset; gap-open and gap-extension penalties 5 and 3) [55] against the *Mus musculus* GRCm39 cDNA reference (Ensembl release 111). Taxonomic classification was performed with Kraken2 (version 2.1.3; confidence threshold 0.5, minimum of two hit groups) [56] against the PlusPF database (May 2025 release; bacterial, archaeal, viral, plasmid, human, protozoan, fungal and UniVec_Core sequences), and species-level abundances were re-estimated with Bracken (version 3.1; read length 150 bp, species level, minimum of 5 reads) [57]. Species-level profiles were exported for downstream analysis in R (version 4.6.1). Relative abundances were analyzed at species level and at genus level, the latter obtained by aggregating species-level estimates. Taxa non-zero in at least 6 of 18 animals were retained and centered log-ratio transformed on the natural logarithmic scale after addition of half the smallest positive value, in accordance with compositional data practice [37]. Shannon diversity and richness were computed from all species. Taxa assigned to *Homo sapiens* and to a *Mus musculus* endogenous provirus were retained in the primary analysis; excluding them changed no result (Tables S4–S6).

### 4.5. Fecal ^1^H-NMR and Untargeted LC-MS Data

Fecal ^1^H-NMR metabolomic profiling of the same animals was performed and published separately [12] and was neither acquired nor re-analyzed as part of the present study; its acquisition and processing parameters are given in that report. No NMR data enter any analysis reported here.

For untargeted metabolomic and lipidomic profiling, approximately 25 mg of cortex, hippocampus or cerebellum, a 25 mg aliquot of the homogenized whole distal colon, or 50 mg of fecal material was extracted by biphasic liquid–liquid extraction in methanol/water/dichloromethane (200, 100 and 300 µL; 2:1:3, *v*/*v*/*v*) with steel beads in a bead beater (two 1 min rounds, on ice between rounds) and centrifuged at 13,000× *g* for 20 min at 4 °C. The upper aqueous phase (polar metabolites) and the lower organic phase (lipids) were collected separately, dried under nitrogen and stored at −80 °C. Aqueous extracts were reconstituted in 200 µL of water/acetonitrile (5:95, *v*/*v*) and organic extracts in 500 µL of water/acetonitrile/isopropanol (1:1:2, *v*/*v*/*v*), centrifuged at 13,000× *g* for 20 min and at 5000× *g* for 10 min, respectively (4 °C), and transferred to Total Recovery vials (Waters). Polar metabolites were separated on an ACQUITY UPLC BEH HILIC column (2.1 × 150 mm, 1.7 µm; Waters) at 35 °C with acetonitrile/water 95:5 (A) and 50:50 (B), both containing 10 mM ammonium acetate and 0.1% formic acid. The gradient was held at 99% A for 2 min, decreased to 45% A at 8 min and 1% A at 9 min (0.4 mL/min), held at 1% A with the flow increased to 0.8 mL/min until 11 min, returned to 99% A at 11.1 min (0.8 mL/min until 19 min) and re-equilibrated at 0.4 mL/min until 23 min; the injection volume was 10 µL. Spectra were acquired on a Xevo G2-XS QTof mass spectrometer (Waters) with electrospray ionization in positive and negative modes (*m*/*z* 50–1000; scan time 0.2 s; capillary voltage 3.0 and 2.5 kV; cone voltage 30 V; source temperature 120 °C; desolvation temperature 400 and 350 °C; desolvation gas 800 L/h), with leucine enkephalin (0.25 ng/µL) as lock mass every 30 s. Lipids were separated on an ACQUITY UPLC CSH C18 column (2.1 × 100 mm, 1.7 µm; Waters) at 55 °C and 0.4 mL/min with acetonitrile/water 60:40 (A) and isopropanol/acetonitrile 90:10 (B), both containing 10 mM ammonium formate and 0.1% formic acid, using a 20 min gradient (57% A at 2 min, 50% at 2.1 min, 46% at 12 min, 30% at 12.1 min and 1% at 18 min, followed by re-equilibration at 60% A; injection volume 3 µL), and acquired in positive-ion MSE mode (*m*/*z* 150–1200; scan time 0.2 s; collision energy ramp 30–40 V; capillary voltage 2 kV; cone voltage 30 V; source temperature 120 °C; desolvation temperature 550 °C; desolvation gas 900 L/h) with leucine enkephalin (2 ng/µL) as lock mass. Autosamplers were kept at 4 °C. Pooled quality-control (QC) samples were prepared separately for the HILIC and C18 analyses by combining aliquots of the corresponding reconstituted extracts [58]; each was injected at least five times to condition the column and then at the beginning of the sequence, after every six study samples and at the end of the run. All samples of each matrix were acquired within a single day in interleaved order across genotype and age, one matrix at a time. Raw data were processed in Progenesis QI v4.1 (Waters) for retention-time alignment, feature detection and intensity extraction. Compounds were annotated against the Human Metabolome Database (HMDB) and LIPID MAPS on the basis of accurate mass, isotope pattern and retention time, and every retained annotation was supported by a match between experimental MSE fragmentation data and database reference spectra; features without a supporting fragment match were excluded, and no separate targeted MS/MS acquisition was performed. Only annotated compounds entered subsequent analyses, and all assignments are reported as putative annotations rather than confirmed identifications. All annotations correspond to level 2 of the Metabolomics Standards Initiative (putatively annotated compounds) [59]; none was confirmed against an authentic standard (level 1). For each age, features were exported from Progenesis QI after filtering for genotype differences (ANOVA *p* < 0.05 and fold change ≥ 1.5). A two-column offset between header and data in the fecal metabolomic table was corrected before analysis. Because these matrices comprise per-age exports of genotype-filtered features, they were analyzed descriptively only and are reported in Table S3.

### 4.6. Statistical Analysis

Genotype effects were estimated by empirical Bayes moderated linear models of abundance on age group and genotype [60,61], with age effects and genotype-by-age interaction assessed by moderated F tests on the corresponding terms, and *p* values adjusted within each view and contrast by the Benjamini–Hochberg procedure [62]. Microbiome effects are reported as differences in centered log-ratio abundance.

Multivariate separability was assessed by a permutational analysis of variance statistic [63] computed on Euclidean distances between standardized complete features for proteomes and on Aitchison distances for microbiome profiles, summed across age strata. Significance was obtained by exact enumeration of all 20^3^ = 8000 within-age genotype assignments, with 2000 assignments for the intestinal proteome where *n* = 16. The observed assignment was included in the enumeration, so *p* values are exact [64]; because the statistic is invariant to reversal of labels within a stratum, the smallest attainable value is 0.001. Shannon diversity, richness and multivariate dispersion were tested in the same framework.

Protein sets were defined from protein identity before testing and comprised microglial and lysosomal, astrocytic, endoplasmic reticulum chaperone, synaptic, myelin, oxidative phosphorylation, tricarboxylic acid cycle and cholesterol synthesis sets. The test statistic was the mean age-adjusted genotype t of quantified members, evaluated against the same 8,000 within-age assignments, with false discovery rate control across all sets and regions; sets with fewer than three quantified members were not tested, which excluded the cholesterol synthesis set in all regions (one quantified member, Fdps, in hippocampus and cortex; two in cerebellum) and the microglial and lysosomal set in cerebellum (one quantified member, Ctsd). Numbers of quantified members per set and region are given in Table S2.

MOFA+ (mofapy2 0.7.5) [16,17] was fitted to the six complete views with Gaussian likelihoods, view scaling, ten initial factors with factors explaining less than 1% of variance pruned during training, automatic relevance determination on weights, no spike-and-slab prior, slow convergence mode and seed 42. Factor scores were regressed on age group and genotype, and the family-wise *p* value for genotype was taken as the proportion of the 8000 assignments whose maximum absolute genotype t across factors equaled or exceeded the observed maximum. Age association was tested by F test and technical association by correlation with per-sample detected feature counts. Robustness was assessed across six random seeds, leave-one-view-out models, brain-only and gut-only models, a top-50% variance filter, a detection threshold of at least 12 of 18 animals, omission of per-sample normalization, and substitution of species for genus profiles, with correspondence between runs quantified as the absolute correlation between genotype-associated factor scores.

Classification used two-component partial least squares discriminant analysis under leave-one-out cross-validation. Within each fold, missing values were imputed from training-fold row minima, the 30 features of largest age-adjusted absolute genotype t were selected using training-fold labels only, and centering and scaling parameters were estimated on the training fold alone, so that the held-out animal contributed to no step of model construction. The null was generated from 999 within-age label assignments drawn without replacement from the 8000 possible assignments, excluding the observed one, and *p* was computed as (1 + the number of null errors ≤ the observed error)/1000, giving a minimum attainable *p* of 0.001. A variant in which feature selection was performed once on all animals was run to quantify optimism [18,19], and the values obtained when imputation was performed outside the cross-validation loop are reported in Table S6 for completeness.

For the individual-level analysis, every feature was centered within its genotype and age cell and Pearson correlations were computed for all cross-view pairs, with the test statistic defined as the percentage of pairs reaching nominal *p* < 0.05 at 11 residual degrees of freedom (|r| > 0.553), the partial-correlation degrees of freedom after removal of the six genotype-by-age cell means (18 − 2 − 5). A variant using the naive threshold for 18 unstructured observations is reported in Table S6. The null was obtained by exact enumeration of all (3!)^6^ = 46,656 within-cell permutations of animal labels in one view, and *p* was the proportion of permutations, including the observed assignment, with a burden at least as large as observed. Power was estimated by planting a shared individual-level signal into a fraction of the features of both views (Table S6). Positive controls comprised the fecal proteome against fecal genera, the three pairwise combinations of brain regions, and an index of blood-derived proteins compared across regions.

Analyses were performed in Python 3.12 with NumPy 2.4, SciPy 1.17, statsmodels 0.15, scikit-learn 1.8 and mofapy2 0.7.5; figures were drawn in R (RStudio 2026.01.0+392) with ggplot2, patchwork and ggrepel.

## 5. Conclusions

5xFAD genotype is associated with reproducible molecular alterations across brain and gut-related compartments of the same animals: a glial and proteostatic reorganization of the brain proteome, a depletion of bile-acid-modifying fecal taxa that cannot be separated from genotype-specific housing, and a shared genotype-associated latent structure linking the two that is statistically robust to exact permutation but minor in magnitude and dependent on preprocessing. That shared axis describes covariance associated with genotype and is not evidence of communication between compartments, a distinction that the accompanying individual-level analysis makes concrete: brain–gut covariation within genotype and age cells did not exceed the permutation null, and neither did same-animal positive controls, which bounds what cohorts of this size can resolve. The findings describe genotype-associated molecular differences across brain and gut compartments of the 5xFAD model, identify a mechanistically tractable microbial hypothesis centered on bile acid modification, and specify the design requirements, cage control, sex balance, absolute quantification and perturbation, under which that hypothesis can be tested.

## Figures and Tables

**Table 1:** Cohort, data views and analytical role.

Cell	Animal IDs	Age (Months)	Notes	
Control-3/5xFAD-3	M04–M06/M01–M03	3	Fecal metagenome of M03 dominated by *Curtobacterium* (65.4%)	
Control-6/5xFAD-6	M10–M12/M07–M09	6	Intestinal proteome missing for M10	
Control-9/5xFAD-9	M16–M18/M13–M15	9	Intestinal proteome missing for M14	
**Data view**		**Features (raw → retained)**	**Missing (%)**	**Role**
Hippocampal proteome		2051 → 2032	2.0	Differential analysis; MOFA+
Cortical proteome		1601 → 1597	1.0	Differential analysis; MOFA+
Cerebellar proteome		1482 → 1473	0.4	Differential analysis; MOFA+
Intestinal proteome (*n* = 16)		1119 → 1099	2.1 (among the 16 animals measured)	Differential analysis; MOFA+
Fecal proteome		1002 → 993	3.1	Differential analysis; MOFA+
Fecal metagenome, genus		197 → 98	0	Compositional analysis; MOFA+
Fecal metagenome, species		468 → 200	0	Sensitivity analysis
Untargeted LC-MS (10 matrices)		32–4267 per matrix	56–65	Post-selection description only

All animals were male. Proteins were collapsed to gene symbols and features detected in fewer than 6 of 18 animals were removed. Complete lists of identified and retained features for every view are provided in Data S1. Controls were wild-type littermates of the 5xFAD animals, and fecal material was collected from each animal individually. Littermates were separated by genotype at weaning and housed in genotype-specific social cages, with each genotype and age group distributed across three cages, with one analyzed animal per cage.

**Table 2:** Principal genotype-associated findings and their evidential status.

Compartment or Analysis	Finding	Effect and Significance	Evidential Status
Hippocampus	Clu, App, Hexb, Apoe, Gfap increased	log2FC +0.72 to +1.31; FDR 7.6 × 10^−4^ to 5.0 × 10^−3^	Directly observed; concordant with published 5xFAD proteomes
Cortex	Fdps, Pdia6, Gja1, Clu, Ctsd, Gfap, Cst3 increased	+0.40 to +1.13; FDR 3.8 × 10^−5^ to 6.7 × 10^−3^	Directly observed
Cerebellum	Apoe, Gfap increased	+0.85, +0.80; FDR 9.4 × 10^−3^, 0.045	Statistical inference
Brain protein sets	Microglial/lysosomal and astrocytic sets elevated in hippocampus and cortex; ER chaperone set elevated in all three regions	mean t +1.16 to +3.98; FDR 0.001–0.029 (ER chaperone FDR ≤ 0.008)	Statistical inference; defines the phenotype as glial and proteostatic
Brain protein sets	Synaptic, myelin, OXPHOS and TCA sets in hippocampus and cortex	No statistically detectable difference (FDR ≥ 0.097)	Absence of a detectable difference, not evidence of no change
Brain protein sets	OXPHOS and TCA sets reduced in cerebellum	mean t −0.94 (FDR 0.042) and −1.90 (FDR 0.011)	Statistical inference; modest effect
Fecal microbiome	Reduced Shannon diversity; *Romboutsia* and *Turicibacter* near-absent; *Lactobacillus* reduced	ΔCLR −7.50, −8.08, −3.39; FDR 1.2 × 10^−8^ to 1.1 × 10^−3^; diversity exact *p* = 0.0015, ΔCLR depends on pseudocount (−6.4 to −8.9)	Statistical inference; cage nested within genotype, so housing effects cannot be excluded
Gut proteomes	Genotype-separable profiles; Lgals6, Mgst2, Stat1 individually significant	FDR 2.1 × 10^−4^ to 0.044	Statistical inference; exploratory
Integration	Genotype-associated latent factor shared across brain and gut views	t = 4.99; exact *p* = 0.00125; ≤ 6.3% variance per view	Statistical inference about covariance, not about communication
Individual-level coupling	Brain–gut correlation burden not above null; same-animal positive controls also null	4.14% vs 4.26% (null median); *p* = 0.66	Bound on design resolution, not evidence of absent coupling

ΔCLR denotes the difference in centered log-ratio abundance on the natural logarithmic scale. ER, endoplasmic reticulum; OXPHOS, oxidative phosphorylation; TCA, tricarboxylic acid; FDR, false discovery rate; log2FC, log2 fold change.

## Data Availability

The mass spectrometry proteomics data have been deposited to the ProteomeXchange Consortium via the PRIDE partner repository (submission reference 1-20261005-122100-1000009832); the dataset identifier and reviewer access credentials will be provided to the Editorial Office as soon as they are issued and before peer review is completed. The metabolomics and lipidomics data have been deposited in MetaboLights under accession MTBLS15920.

## Supplementary Materials

The following supporting information can be downloaded at https://www.mdpi.com/article/10.3390/ijms27219879/s1.
