# Supplementary material for: Genotype-Associated Molecular Phenotypes Across Brain and Gut Compartments in Male 5xFAD Mice: Shared Latent Structure and the Limits of Individual-Level Inference
Source: Int J Mol Sci. 2026 Nov 4;27(21):9879. doi: 10.3390/ijms27219879 (PMC13763930; doi:10.3390/ijms27219879)
Supplement: Supplementary file 1 [file ijms-27-09879-s001.zip › supplementary figures.pdf]

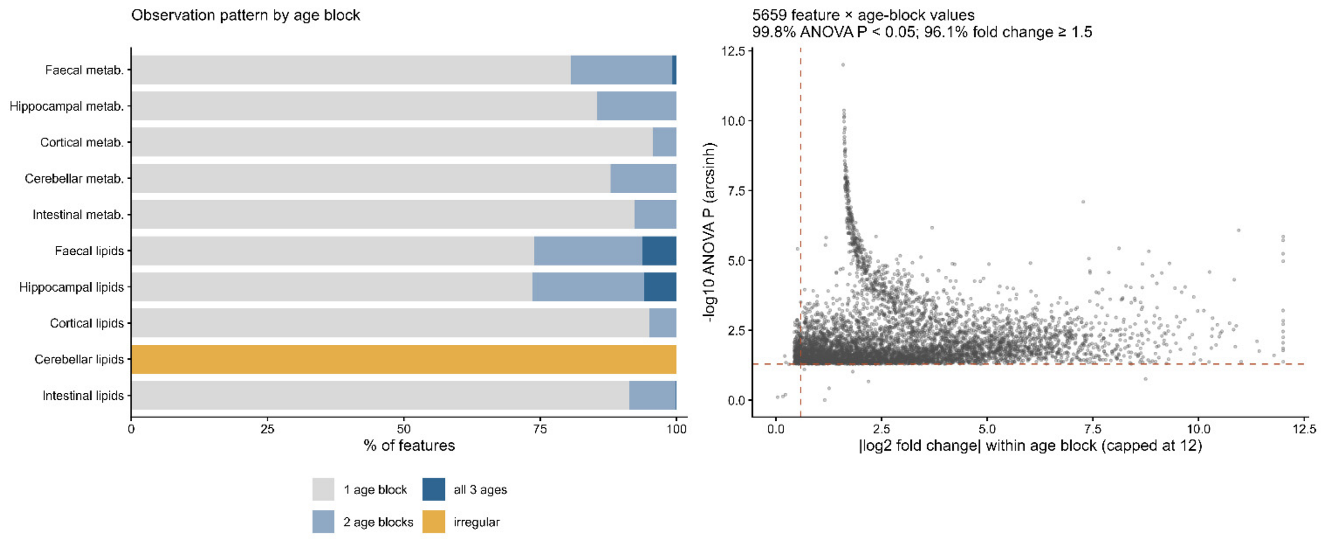

Supplementary Figure S1

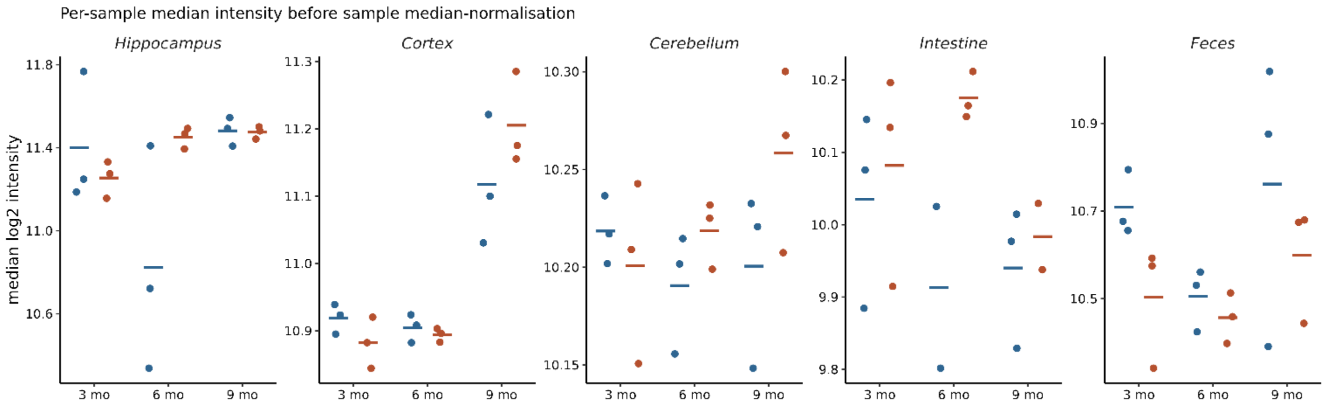

Supplementary Figure S2

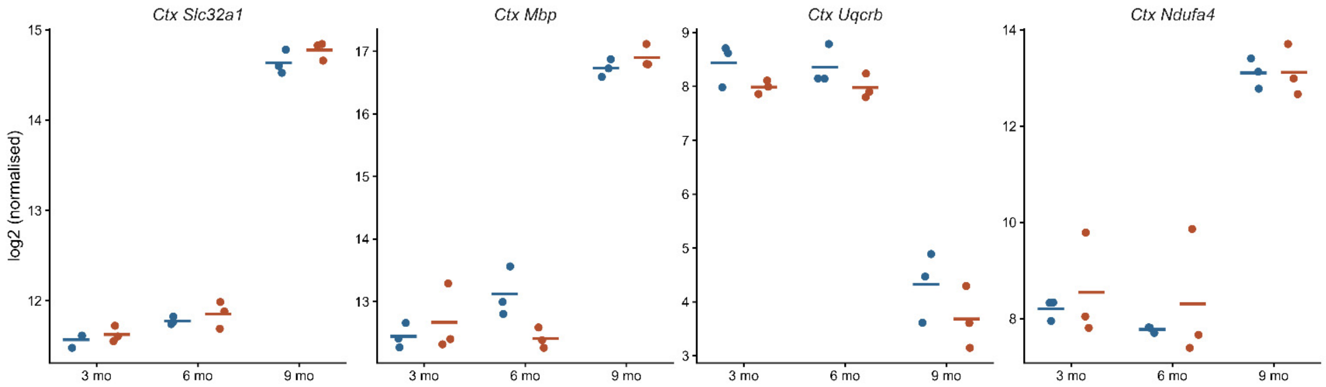

Supplementary Figure S3

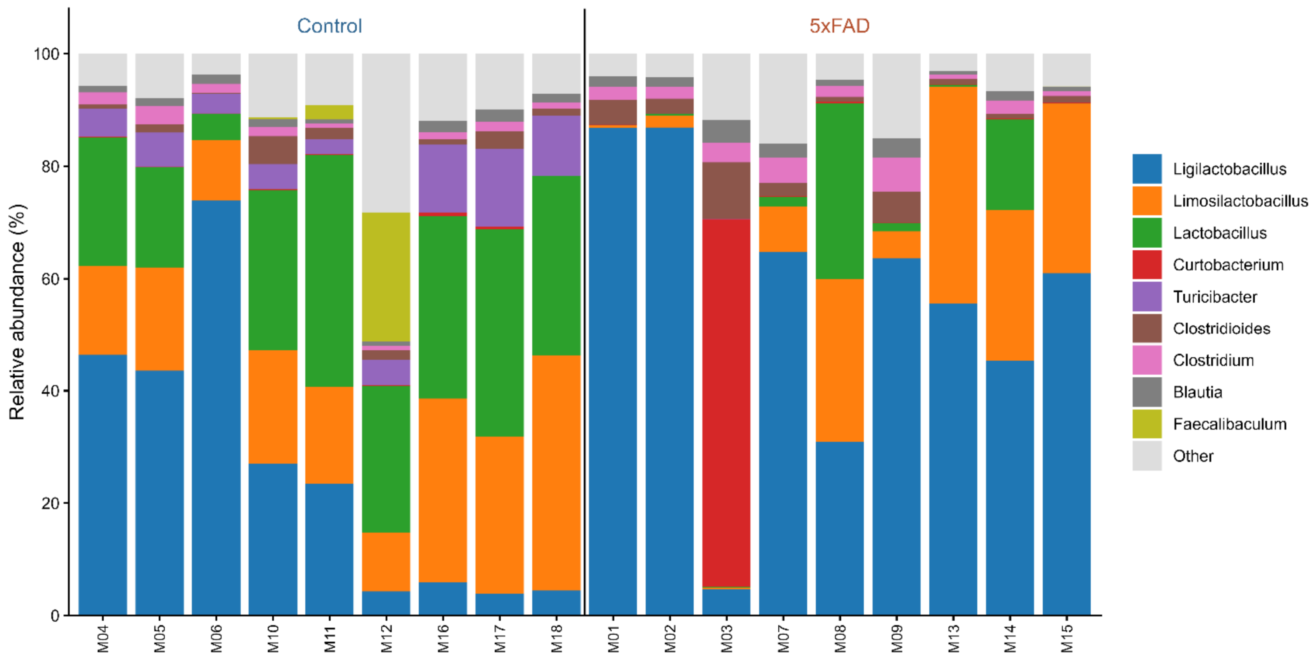

Supplementary Figure S4

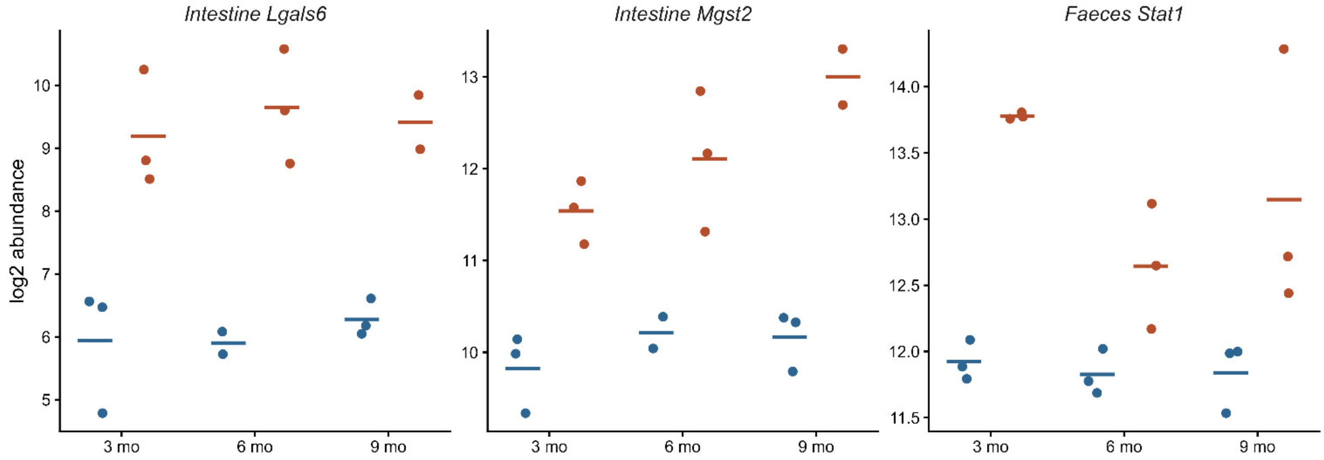

Supplementary Figure S5

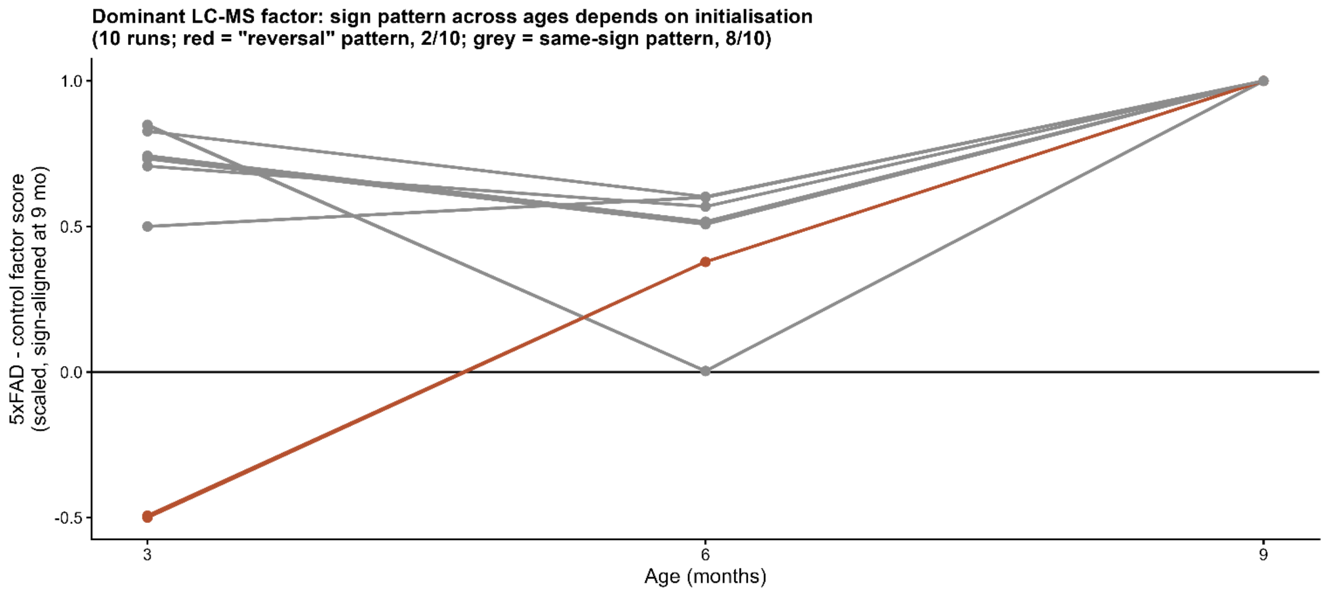

Supplementary Figure S6
